# AIMp1 Potentiates T_H_1 Polarization and Is Critical for Effective Antitumor and Antiviral Immunity

**DOI:** 10.3389/fimmu.2017.01801

**Published:** 2018-01-15

**Authors:** Dan Liang, Lin Tian, Ran You, Matthew M. Halpert, Vanaja Konduri, Yunyu C. Baig, Silke Paust, Doyeun Kim, Sunghoon Kim, Fuli Jia, Shixia Huang, Xiang Zhang, Farrah Kheradmand, David B. Corry, Brian E. Gilbert, Jonathan M. Levitt, William K. Decker

**Affiliations:** ^1^Department of Pathology and Immunology, Baylor College of Medicine, Houston, TX, United States; ^2^Department of Molecular and Cellular Biology, Baylor College of Medicine, Houston, TX, United States; ^3^Department of Pediatrics, Texas Children’s Hospital, Houston, TX, United States; ^4^Center for Human Immunobiology, Texas Children’s Hospital, Houston, TX, United States; ^5^Department of Molecular Virology and Microbiology, Baylor College of Medicine, Houston, TX, United States; ^6^Dan L. Duncan Cancer Center, Baylor College of Medicine, Houston, TX, United States; ^7^Medicinal Bioconvergence Research Center, Department of Molecular Medicine and Biopharmaceutical Sciences, Seoul National University, Seoul, South Korea; ^8^Antibody-based Proteomics Core, Baylor College of Medicine, Houston, TX, United States; ^9^Lester and Sue Smith Breast Center, Baylor College of Medicine, Houston, TX, United States; ^10^Division of Pulmonary, Critical Care, and Sleep Medicine, Baylor College of Medicine, Houston, TX, United States; ^11^Division of Immunology, Allergy, and Rheumatology, Baylor College of Medicine, Houston, TX, United States; ^12^Scott Department of Urology, Baylor College of Medicine, Houston, TX, United States; ^13^Center for Cell and Gene Therapy, Baylor College of Medicine, Houston, TX, United States

**Keywords:** dendritic cell, AIMp1, T_H_1 immunity, IL-12, antitumor immunity, antiviral immunity, p38 MAPK signaling

## Abstract

Dendritic cells (DCs) must integrate a broad array of environmental cues to exact control over downstream immune responses including T_H_ polarization. The multienzyme aminoacyl-tRNA synthetase complex component AIMp1/p43 responds to cellular stress and exerts pro-inflammatory functions; however, a role for DC-expressed AIMp1 in T_H_ polarization has not previously been shown. Here, we demonstrate that the absence of AIMp1 in bone marrow-derived DC (BMDC) significantly impairs cytokine and costimulatory molecule expression, p38 MAPK signaling, and T_H_1 polarization of cocultured T-cells while significantly dysregulating immune-related gene expression. These deficits resulted in significantly compromised BMDC vaccine-mediated protection against melanoma. AIMp1 within the host was also critical for innate and adaptive antiviral immunity against influenza virus infection *in vivo*. Cancer patients with AIMp1 expression levels in the highest tertiles exhibited a 70% survival advantage at 15-year postdiagnosis as determined by bioinformatics analysis of nearly 9,000 primary human tumor samples in The Cancer Genome Atlas database. These data establish the importance of AIMp1 for the effective governance of antitumor and antiviral immune responses.

## Introduction

Of the professional antigen-presenting cell (APC) subsets, dendritic cells (DCs) are the most specialized and efficient in the priming of *de novo* T-cell responses and thus serve as a critical bridge between innate and adaptive immunity. In this sentinel capacity, DC must detect, process, and integrate a broad array of environmental cues to generate downstream responses best-tailored to specific pathogenicities. A critical aspect of this process involves regulation of T-helper (T_H_) cell polarization. T_H_ polarization, in turn, is canonically informed by a vast array of pattern recognition, cytokine, chemokine, costimulatory, and other receptor complexes (1–3). T-helper type 1 (T_H_1) polarization is associated with the generation of cell-mediated adaptive responses provided by effector cells including CD4^+^ T helper 1 (T_H_1) cells and CD8^+^ cytotoxic T lymphocytes (CTLs) and is characterized by the secretion of IL-12 and IFN-γ from APC and T-cells, respectively (4). These types of adaptive responses are known to be critical for effective clearance of intracellular infection and well correlated with positive outcomes in cancer (5–7). Indeed, recent novel approaches in cancer immunotherapy including vaccines (8–10) and engineered T-cells (11) have been correlated with clinical benefit when hallmarks of T_H_1 immunity are observed. Successful implementation of these approaches can be further enhanced by administration of immune checkpoint inhibitors (12); however, consistent generation of robust and durable T-cell immunity remains an elusive goal in many patients. Therefore, interrogation of the critical factors that govern the T_H_1 immune response enhance the effort to manipulate adaptive immunity for medical benefit (13, 14).

AIMp1/p43 is a structural component of the multienzyme aminoacyl-tRNA synthetase (mARS) complex, a large molecular complex comprised of eight aminoacyl-tRNA synthetases arrayed in dimeric fashion and bound together by core structural proteins. Though the primary functions of this protein complex remain largely uncharacterized, AIMp1 is known to be released from the mARS complex and secreted under certain conditions including cellular stress (15–17). In addition, recent work indicates that other mARS components can dissociate from this complex upon viral infection and interact with critical components of innate antiviral immunity (18). Genetic ablation of AIMp1 enhances T_H_2-biased airway hyperreactivity in a model of allergic airway inflammation (19), and upregulated AIMp1 gene expression was recently identified as part of a good-prognosis gene signature in glioblastoma multiforme (20). *In vitro* studies have shown that recombinant *Escherichia coli*-expressed AIMp1 protein can upregulate IL-12 secretion from bone marrow-derived macrophages and DC in an NF-κB-dependent fashion, enhancing the generation of IFN-γ-secreting CD4^+^ T-cells (21, 22). Recombinant AIMp1 also induces B-cell activation and proliferation accompanied by increased class switch recombination toward the T_H_1-specific IgG_2_ isotype and antigen-specific antibody production (23). AIMp1 also functions through macrophages to activate NK cells both *in vitro* and *in vivo* (24). These findings suggest a positive link between AIMp1 and T_H_1 immunity, but there remains a lack of direct *in vivo* and/or cell type-specific evidence to determine the validity of this hypothesis. Further, no study has yet demonstrated a necessity for DC-expressed AIMp1 to regulate T_H_1 polarization, specifically in the *in vivo* antitumor/antiviral setting, nor addressed the cellular and/or molecular mechanisms required for its proper function.

Here, we demonstrate that bone marrow-derived DC (BMDC)-expressed AIMp1 is critical to the propagation of T_H_1 responses in antitumor immunity, at least partly through positive regulation of the p38 MAPK signaling pathway. Microarray analysis indicates AIMp1 impacts the transcription of hundreds of genes and multiple biological and immunological processes within BMDC, including innate antiviral responses. The importance of AIMp1 to T_H_1 antiviral and antitumor immunity was also demonstrated by *in vivo* model systems of melanoma and influenza virus infection as well as analysis of the nearly 9,000 primary human tumors in The Cancer Genome Atlas (TCGA) database to which outcomes data could be linked. These data identify an important role for BMDC-expressed AIMp1 in the positive regulation of T_H_1 immunity and provide significant insights into the manner by which DC regulate adaptive immune responses.

## Materials and Methods

### Mice

The AIMp1 null allele in the C57BL/6 background was graciously provided by Dr. Sunghoon Kim at the Seoul National University. Influenza experiments were performed in 129Sv/Ev mice into which the null allele had been backcrossed to the F_7_ generation. Tumor experiments were performed in C57BL/6×129Sv/Ev F_1_ heterozygotes. All AIMp1 deficient animals were derived from heterozygous breeders, and wild-type (WT) control populations in all experiments were always derived from the *littermates* of those intercrosses. Other mice utilized included WT C57BL/6J, 129Sv/Ev and OT-II were purchased from the Jackson Laboratory (Barr Harbor, ME, USA). Experiments were performed utilizing NIH and United States Department of Agriculture guidelines, The Public Health Service Policy on Humane Care and Use of Laboratory Animals, and experimental protocols approved by the Baylor College of Medicine Investigational Animal Care and Use Committee (IACUC Protocol numbers AN-1428 and AN-2307).

### Reagents

p38 MAPK inhibitors SB202190 and SB203580 (Sigma-Aldrich, St. Louis, MO, USA), PP2A inhibitors Okadaic acid (Sodium Salt, EMD Millipore, Billerica, MA, USA) and Endothall (Sigma-Aldrich), DUSP1/6 Inhibitor (BCI, EMD Millipore); OVA (257–264)/SIINFEKL peptide (Anaspec, Fremont, CA, USA), recombinant endotoxin-free OVA protein (Invivogen, San Diego, CA, USA), Toll-like receptor (TLR) agonists LPS (O127:B8 *E. coli* strain, Sigma-Aldrich), and mouse IL-6 ELISA and mouse IL-1β ELISA sets (BD Pharmingen, San Diego, CA, USA). Other antibodies and protein standards were purchased from Biolegend including purified antimouse IL-12 (p70) antibody (C18.2), biotin antimouse IL-12/IL-23 p40 antibody (C17.8), recombinant mouse IL-12 (p70) (ELISA Std.), purified antimouse IFN-γ antibody (AN-18), biotin antimouse IFN-γ antibody (R4-6A2), recombinant mouse IFN-γ (ELISA Std.), purified antimouse IL-4 antibody (11B11), and biotin antimouse IL-4 antibody (BVD6-24G2).

### Generation of Murine BMDC

Bone marrow leukocytes were flushed from mouse tibia and femur and cultured in RPMI-1640 (Lonza, Allendale, NJ, USA) containing 10% fetal bovine serum (Invitrogen, Carlsbad, CA, USA), 1% antibiotic–antimycotic (Invitrogen) and supplemented with 20 ng/mL mouse GM-CSF (R&D Systems, Minneapolis, MN, USA) and 10 ng/mL mouse IL-4 (R&D Systems). Cells were cultured in a humidified chamber at 37°C and 5% atmospheric CO_2_. On day 3, half the culture medium was gently removed and replenished with fresh medium and cytokines. BMDC were harvested on day 6 with Cell Dissociation Buffer (Invitrogen). The immature BMDC were then treated with lipopolysaccharide (Sigma-Aldrich) or maturation cytokine cocktail comprising 10 ng/ml IL-1β (R&D Systems), 10 ng/ml TNF-α (R&D Systems), 15 ng/ml IL-6 (R&D Systems), and 1 µg/ml PGE_2_ (Sigma-Aldrich).

### Murine BMDC Antigen Loading

Immature BMDC were tandemly loaded with peptide and protein or with tumor cell-derived mRNA and lysate as described previously (25). Briefly, cells were resuspended in Viaspan (Barr Laboratories, Pomona, NY, USA) buffer at 40 × 10^6^ cells/ml and incubated with mRNA (1 µg mRNA/10^6^ cells) or peptide (10 µg/ml) on ice for 10 min. Cells were then electroporated with exponential decay pulse (250 V, 125 μF, Ω = ∞) using a Gene Pulser-X cell (Bio-Rad, Hercules, CA, USA) in Gene Pulser 4 mm cuvettes (Bio-Rad). Shortly after electroporation, cells were supplemented with RPMI-1640 (5% FBS) and incubated with lysate or protein antigen (10 µg/ml). At this stage, peptide-loaded cells were also supplied with more peptide to reach a final concentration of 10 µg/ml. After 3 h of antigen loading, cells were gently washed with PBS and cultured in fresh RPMI-1640 (10% FBS) containing maturation cytokine cocktail as described above for either 24 h (*in vivo* experiments) or 48 h (*in vitro* experiments). All unloaded control groups received electroporation without antigens under identical culture conditions (*mock-electroporation*).

### *In Vitro* Mouse CD4^+^ T-Cell Differentiation

Naive splenic CD4^+^ T-cells were isolated using anti-CD4-conjugated magnetic beads (Miltenyi Biotec, San Diego CA, USA) with an autoMACS cell separator. CD4^+^ splenic T-cells were differentiated under T_H_1 polarizing conditions. Briefly, 2.0–2.5 × 10^6^/ml cells were activated with 1.5 µg/ml plate-coated anti-CD3 (BD Pharmingen) and 1.5 µg/ml soluble anti-CD28 antibodies (BD Pharmingen) in addition to 10 µg/ml anti-IL-4 blocking antibody, 50 U/ml IL-2 and 20 ng/ml IL-12. Cells were cultured for 3–5 days, then harvested and washed for intracellular staining of IFN-γ.

### DC and T-Cell Coculture

OT-II or naive T-cells from mouse splenocytes were enriched by Pan T Cell Negative Isolation Kit (Miltenyi Biotec). Purified T-cells were cultured together with BMDC at a ratio of 10:1 unless specified otherwise in 96-well U-bottomed tissue culture plates with RPMI-1640 supplemented with 10% FBS. The medium and cells were collected on day 3 for further analysis.

### Quantitative Realtime Reverse Transcription PCR (qRT-PCR)

Total RNA from cell lines or mouse bronchoalveolar lavage fluid (BALF) was extracted with QIAzol lysis reagent (Qiagen, Germantown, MD, USA); total RNA from mouse lung tissue was extracted with RNeasy Mini Kit (Qiagen) according to the manufacturer’s instructions. cDNA was synthesized with the high-capacity cDNA reverse transcription kit (Life Technologies, Carlsbad, CA, USA) according to the manufacturer’s instructions. qRT-PCR was performed using a 7500 Realtime PCR system (Applied Biosystems, Foster City, CA, USA) with the Taqman Realtime PCR assay (ThermoFisher Scientific, Waltham MA, USA) according to the manufacturer’s instructions. Primers: il6 (Mm00446190_m1, FAM), il12a (Mm00434165_m1, FAM), il12b (Mm01288989_m1, FAM), ifng (Mm01168134_m1, FAM), ifit1 (Mm00515153_m1, FAM), mx2 (Mm00488995_m1, FAM), oasl1 (Mm00455081_m1, FAM), irf7 (Mm00516793_g1, FAM), ifna1 (Mm03030145_gH, FAM), and 18s rRNA (4319413E, VIC).

### Western Blotting

Preparation of whole cell lysate: cells were lysed in 1% NP-40 lysis buffer containing protease inhibitor cocktail, phosphatase inhibitor cocktail 2 and phosphatase inhibitor cocktail 3 (all purchased from Sigma-Aldrich) on ice with vortexing every 10–15 min. Cell lysate was centrifuged at 14,000 *g* for 15 min and the cleared lysate was denatured with laemmli buffer (Bio-Rad) containing 5% β-mercaptoethanol (Bio-Rad) for 10 min. Denatured whole cell lysate samples were stored at −20°C for further analysis. Electrophoreses and blotting: proteins samples were separated by SDS-gel electrophoreses (Invitrogen) with subsequent transfer to a 0.45 µm nitrocellulose membrane (Bio-Rad) for antibody probing. All blocking and antibody staining steps were carried out in 5% BSA (RPI, Grainger) in 1 × TBST buffer (0.05% Tween-20). Western blotting chemiluminescent signal was detected with SuperSignal West Femto Maximum Sensitivity Substrate (ThermoFisher Scientific) using a ChemiDoc XRS digital imaging system supported by Image Lab software version 2.0.1 (Bio-Rad). Densitometry was performed using Image Lab software. Antibodies used for Western blotting: antihuman/mouse AIMp1 (Lifespan Biosciences Inc., Seattle, WA, USA); phospho-STAT1(Tyr701) (58D6), STAT1, phospho-STAT4(Tyr693) (D2E4), T-bet/TBX21 (V365), phosphor-IKKα/β (Ser176/180) (16A6), phospho-p38 MAPK(Thr180/Tyr182) (D3F9), p38, phospho-SAPK/JNK (Thr183/Tyr185) (G9), phospho-p44/42 MAPK (ERK1/2)(Thr202/Tyr204) (D13.14.4E), phospho-MKK3(Ser189)/MKK6 (Ser207) (D8E9), MAPKAPK-2 Antibody Sampler Kit, and the PP2A Antibody Sampler Kit were all purchased from Cell Signaling Technology (Danvers, MA, USA); DUSP1/MKP1 (V-15, Santa Cruz, Dallas, TX, USA); antihuman/mouse β-actin (Santa Cruz). Unedited Western blotting gel images can be found in supplementary figures (Figures S7 and S8 in Supplementary Material).

### Flow Cytometry

All flow cytometric analysis was performed using an LSR II flow cytometer (BD Biosciences) and analyzed with FlowJo version 10.0.7 (Tree Star Inc., Ashland, OR, USA). Antibodies used for flow cytometry: eFluor^®^ 450 antimouse CD3ε (145-2C11, eBioscience, San Diego, CA, USA), PE-Cy7 antimouse NK1.1 (PK136, BD Pharmingen), PE-Cy7 antimouse CD19 (1-D3, BD Pharmingen), PE antimouse CD4 (GK1.5, TONBO Biosciences, San Diego, CA, USA), FITC antimouse CD8α (53-6.7, BD Pharmingen), APC antimouse IFN-γ (XMG1.2, TONBO Biosciences), Pacific Blue antimouse CD11c (N418, Biolegend, San Diego, CA, USA), FITC antimouse CD86 (GL1, BD Pharmingen), PE antimouse CD40 (3/23, BD Pharmingen), PE antimouse I-A^b^ (AF6-120.1, BD Pharmingen), PE-Cy5 antimouse CD11b (M1/70, TONBO Biosciences), PE-Cy7 antimouse CD8α (53-6.7, TONBO Biosciences), and APC-Cy7 antimouse CD103 (2E7, Biolegend).

### Tumor Inoculation and Vaccination

Mice were subcutaneously inoculated with 50,000 WT B16F10 melanoma tumor cells (American Type Culture Collection, Manassas, VA, USA) or 200,000 B16F0-OVA melanoma tumor cells (26) resuspended in 100 µl PBS on day 0. On the day of vaccination, mice were allocated based on tumor size. Tumor sizes of each animal were recorded and randomized so that each group possessed similar average tumor sizes and standard errors. 200,000 BMDC loaded with tumor antigen resuspended in 50 µl PBS were injected in the footpad. On boost days, the same number of cryopreserved BMDC were washed and injected in the same fashion as the primary vaccination. Tumor antigens used to vaccinated against B16F10 tumor were B16F10 melanoma tumor-derived mRNA and lysate (as described above). Tumor antigens used to vaccinate against B16F0-OVA tumor was SIINFEKL peptide + recombinant OVA protein (also described above). Tumor size was determined by external caliper measurement and calculated by means of the formula (Length × Width^2^) × π/6.

### Tumor Tissue Dissociation

Mice inoculated with B16F0-OVA tumor cells and vaccinated/boosted with BMDCs on postinoculation day 19 were sacrificed, and tumor tissues were harvested for dissociation with the Tumor Dissociation Kit (mouse, Miltenyi Biotec) according to the manufacturer’s instructions. Briefly, melanoma tumors were cut into small pieces and transferred to the gentle MACS C-tube (Miltenyi Biotec) containing proper enzyme mix (enzyme D, R, A in RPMI serum-free media). C-tubes were loaded onto the gentleMACS Dissociator (Miltenyi Biotec) and processed by programs “m_impTumor_02,” 40 min at 37°C shaker, and “m_impTumor_03.” Cells were collected *via* 70-µm cell strainer followed by red blood cell (RBC) lysis.

### Influenza Virus Infection

Mice were challenged with influenza A/HongKong/8/68 (H3N2) Swiss mouse lung adapted strain of H3N2 influenza A virus graciously provided by Dr. Brian Gilbert (27). Infection was performed by a nebulized 20-min aerosol (Aerotech II nebulizer flowing at 10 L/min of room air generated from an Aridyne 2000 compressor) exposure of influenza virus diluted in MEM media containing 0.05% gelatin. All mice infected for any given experiment were infected simultaneously in a single exposure chamber. Following infection, mice were housed in Baylor College of Medicine’s biohazard facility.

### Bronchoalveolar Lavage (BAL) Fluid Analysis

Mice were anesthetized and BAL fluid was collected by instilling and withdrawing 0.8 ml of sterile PBS twice through the trachea. Total and differential cell counts in the BAL were determined with the standard hemocytometer and HEMA3 staining (Biochemical Sciences Inc., Swedesboro, NJ, USA) of BAL cells on cytospin slides.

### Intracellular Cytokine Staining

Mouse lung RBC-free single cell suspensions were stimulated with 10 ng/ml phorbol 12-myristate 13-acetate (PMA; EMD Millipore) and 1 µg/ml ionomycin (Calcium Salt; ThermoFisher Scientific) overnight and supplemented with 10 µg/ml brefeldin A (eBioscience) for 5 h the following morning. Cells were stained for surface markers with anti-CD3, anti-CD4, and anti-CD8 antibodies and then fixed/permeabilized for intracellular staining of IFN-γ using the Cytofix/Cytoperm Kit (BD Biosciences) according to the manufacturer’s instructions.

### Serum Antibody Isotyping

Serum anti-influenza HA antibody isotypes were quantitated with the Mouse Monoclonal Antibody Isotyping Reagents (Sigma-Aldrich) according to the manufacturer’s instructions. Briefly, polystyrene multiwell plates were coated with 1 µg/ml influenza HA protein (A/Hongkong/1968 hemagglutinin protein, Sino Biological, Beijing, China) and incubated in immune sera diluted 10,000-fold in PBS for 2 h at room temperature. After stringent washing, immune complexes were incubated with the provided anti-isotype specific reagents and detected with peroxidase conjugated rabbit antigoat IgG.

### Cancer Database Survival Analysis

To analyze human *AIMP1* expression across multiple cancer types, we used the TCGA melanoma RNA-seq dataset (dataset ID: TCGA_SKCM_exp_HiSeqV2), TCGA ovarian cancer RNA-seq dataset (dataset ID: TCGA_OV_exp_HiSeq), and pan-cancer RNA-seq dataset (dataset ID: HiSeqV2_PANCAN). Patient sample characteristics were downloaded from the UCSC cancer browser (https://genome-cancer.ucsc.edu/proj/site/hgHeatmap/) (28). Cox regression *p*-values and log rank *p*-values were calculated using “survdiff” command in the “survival” package of R. Kaplan–Meier curves were drawn with the “survfit” command in the same R package by tertiles of *AIMP1* expression. The numbers in parentheses indicate the fraction of the patient samples that could be linked to outcomes data. Analysis of IFN-γ expression was performed using the same methodology.

### Immune Signaling Prediction

To predict the relative percentages of 22 different immune cells in basal-like breast cancer samples from GSE76275 (29), we downloaded data using the “GEOquery” package of R and used the gene expression profiling as the input for CIBERSORT (30, 31) enabling identification of 22 different immune cell signatures as previously described (30). The quantile normalization was disabled as the samples were from the same GEO data set. To generate the signaling score for T-cell subsets, the subset T-cell signature genes were summed (32).

### Reverse Phase Protein Array (RPPA)

Reverse phase protein array was performed at the antibody-based Proteomics Core of the Dan L. Duncan Comprehensive Cancer Center, Baylor College of Medicine. Cell lysate from BMDCs was isolated according to core facility protocol by the investigators. A detailed description of sample processing, antibody validation, and data analysis are available on the facility’s website (https://www.bcm.edu/centers/cancer-center/research/shared-resources/cprit-cancer-proteomics-and-metabolomics/reverse-phase-proteinarray). RPPA is performed as described (33). Heatmaps of log2-transformed data were generated with Cluster 3.0 (http://bonsai.hgc.jp/~mdehoon/software/cluster/) as a hierarchical cluster using Pearson correlation and a centered metric visualized and presented with Treeview software (http://taxonomy.zoology.gla.ac.uk/rod/treeview.html).

### Microarray

Gene expression profiling of BMDC was performed with Affymetrix Mouse Transcriptome Array 1.0 Chip (Affymetrix, Santa Clara, CA, USA) by the Sequencing and Microarray Facility, at the University of Texas MD Anderson Cancer Center (Houston, TX, USA). Total mRNA from BMDCs was isolated by RNeasy Mini Kit (Qiagen) according to the manufacturer’s instructions. A detailed description of sample requirements and data preanalysis is available on the facility’s website (https://www.mdanderson.org/research/research-resources/core-facilities/sequencing-and-microarray-facility-smf/services-and-fees/microarray-services-overview.html). Data were analyzed and visualized with Transcriptome Analysis Console v3.0 (Affymetrix). Gene ontology analysis was performed with WEBGESTALT online resource. Information and raw array data can be found at GEO (GSE102282).

### Statistical Analysis

Significance of differences was determined by two-way analysis of variance (ANOVA) or one-way ANOVA using the Bonferroni *post hoc* test for multiple comparisons unless indicated otherwise. Kaplan–Meier survival curve significance was determined by the log rank (Mantel-Cox) test. Bioinformatic statistics were described in above method section accordingly (*Cancer Database Survival Analysis* and *Immune Signaling Prediction*). All data are displayed as the mean ± SEM, and all analyses were performed using the Prism software (GraphPad Software) unless indicated otherwise. Statistical significance was defined as *p* ≤ 0.05.

## Results

### AIMp1 Is Critical for BMDC Vaccine-Mediated Protection against Melanoma

**Figure 1:**
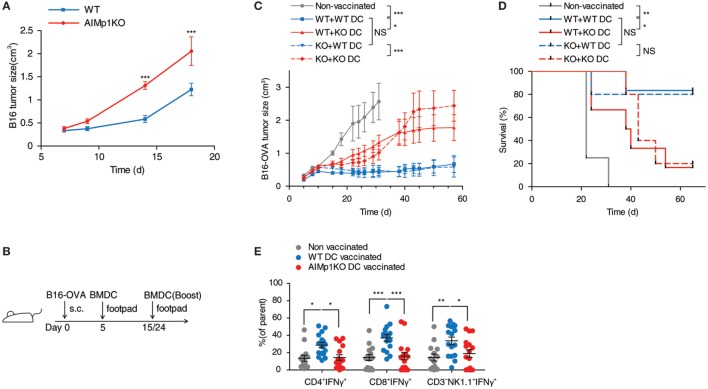
AIMp1 is critical for bone marrow-derived dendritic cell (BMDC) vaccine-mediated control of tumor growth. **(A)** Wild-type (WT) or AIMp1 KO animals were challenged with 50,000 B16-F10 melanoma tumor cells s.c. on day 0 and tumor sizes were measured by caliper (*n* = 5). **(B)** WT or AIMp1 KO animals were challenged with 200,000 B16-OVA melanoma tumor cells s.c. on day 0. On day 5, mice were vaccinated in the footpad with 200,000 WT or AIMp1 KO BMDC loaded with SIINFEKL peptide and OVA protein. On days 15 and 24, mice were boosted in the footpad with an additional 200,000 BMDC. **(C)** Tumor sizes were measured by caliper (*n* = 5–6, representative of two independent experiments). **(D)** Kaplan–Meier survival analysis of animals in **(C)**. *p < 0.05, **p < 0.01, ***p < 0.001 as determined by log rank (Mantel-Cox) test. **(E)** Tumor tissues from animals treated as in **(C)** were harvested on day 19 for lymphocyte isolation and intracellular flow cytometry analysis (*n* = 17, pooled from three independent experiments). Single cell lymphocytes were gated on CD45^+^ and further analyzed for IFN-γ^+^ populations in different populations. Data are displayed as mean ± SEM for **(C,E)**. *p < 0.05, **p < 0.01, ***p < 0.001 as determined by paired two-way analysis of variance (ANOVA) **(C)** and regular two-way ANOVA **(E)** with Bonferroni *post hoc* test for multiple comparisons.

Antitumor immunity is broadly dependent upon T_H_1 immune processes for destruction of neoplastic cells (34–36), and immunity to B16 melanoma is specifically known to be dependent upon T_H_1 T-cell generation (37). Administration of recombinant *E. coli*-expressed human AIMp1 protein to tumor-bearing mice has been shown to have tumor inhibitory effects in a human xenograft model of stomach cancer (38, 39) as well as EG7 lymphoma (40), breast cancer (41), and others. To determine if intrinsic AIMp1 might possess inherent antitumor properties under conditions of physiologic homeostasis, we inoculated AIMp1^−/−^ mice with B16F10 melanoma and observed a tumor growth rate nearly double that exhibited in WT animals (Figure 1A). To investigate if the antitumor effect of AIMp1 relied upon activation of certain immune cell subsets (i.e., DC), we took advantage of the well-characterized B16-OVA tumor cell line which expresses the model antigen ovalbumin (OVA) (26, 42). Cohorts of WT and AIMp1^−/−^ mice were inoculated subcutaneously (s.c.) with 200,000 tumor cells and subsequently vaccinated with BMDC loaded with recombinant OVA protein and its immunodominant MHC class I peptide epitope SIINFEKL (Figure 1B). One WT and one AIMp1^−/−^ cohort were vaccinated with WT BMDC, while one WT and one AIMp1^−/−^ cohort were vaccinated with AIMp1^−/−^ BMDC. A non-vaccinated WT cohort was included as a control. Tumor growth was equally well controlled in both WT and AIMp1^−/−^ cohorts so long as the antigen-loaded BMDC were derived from WT mice. In contrast, tumor growth was poorly controlled in both WT and AIMp1^−/−^ cohorts vaccinated with AIMp1^−/−^ BMDC, ultimately reaching comparable size as non-vaccinated controls with only a short delay (Figure 1C). The trend was well reflected by Kaplan–Meier survival analysis (Figure 1D). These results indicated that BMDC-expressed AIMp1 is critical for vaccine-mediated rejection of immunogenic melanoma tumor, whereas the impact of AIMp1 in host effector cells appeared to have little relevance in this regard. WT tumor recipient mice treated as in this experiment were sacrificed on day 19 and tumor tissues were dissociated for immune cell analysis. Administration of WT BMDC vaccines upregulated the infiltration of IFN-γ^+^ T-cells and NK cells within the tumor, whereas infiltration of IFN-γ^+^ cells among animals vaccinated with AIMp1^−/−^ BMDC was identical to that of unvaccinated mice (Figure 1E). The data indicated that AIMp1 presence within the BMDC vaccine promotes type 1 polarization of tumor infiltrating lymphocytes *in vivo*.

AIMp1 is known to be released under conditions of cell stress, and recombinant AIMp1 protein exerts function directly or indirectly on multiple cell types including APCs, NK cells, and T-cells. Thus, it is possible that any AIMp1 released from BMDC vaccines during the antigen loading and maturation process also exerted pro-inflammatory effects *in vivo* that accounted for its antitumor potential. To control for this concern, mice were vaccinated with *mock-electroporated* WT DC treated identically to loaded BMDC but without exposure to antigen. In this experiment melanoma tumor growth in recipient mice vaccinated with WT unloaded BMDCs (*mock-electroporated*) was nearly identical to that of mice vaccinated with AIMp1^−/−^ antigen-loaded BMDC (Figure S1 in Supplementary Material). These data indicated that AIMp1 released from BMDC during vaccine preparation and injection could not account for any observed differential in antitumor effects. To further determine whether results could be due to differences in BMDC lineage development in the absence of AIMp1, WT and AIMp1^−/−^ BMDC populations were carefully characterized by flow cytometry. This analysis indicated that both populations of BMDC were almost uniformly CD11c^+^ with similar numbers of CD11b^medium^MHC Class II^high^ DC (43) as well as CD8α^+^CD103^+^ cross-presenting DC (44) whether immature, or matured (Figure S2 in Supplementary Material). The absence of observed differences in lineage marker expression suggested that AIMp1^−/−^ BMDC were the lineage equivalent of WT BMDC and that AIMp1 function was more likely to be upon immune regulation rather than upon BMDC development.

### AIMp1 Deficiency in BMDC Impairs T_H_1 Polarization *In Vitro*

**Figure 2:**
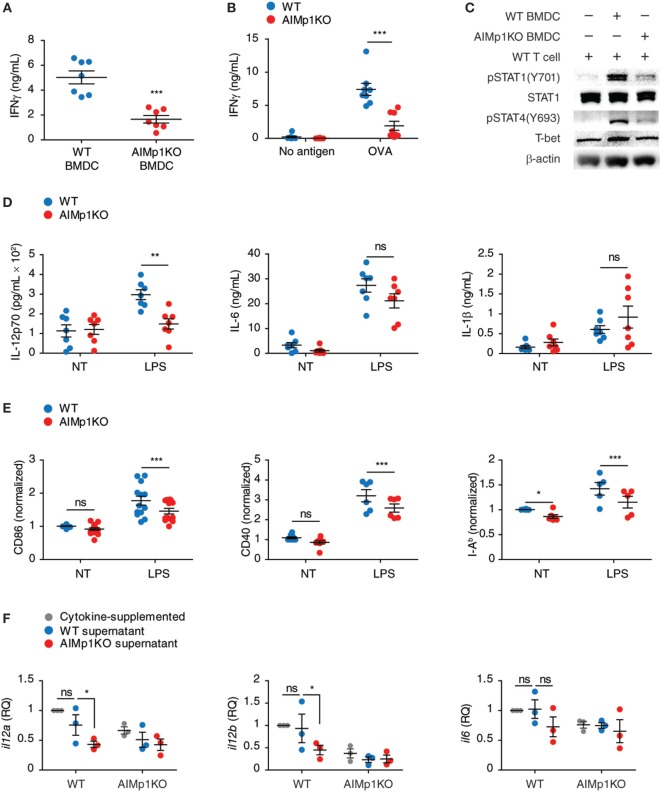
AIMp1 deficiency in dendritic cells impairs T_H_1 polarization *in vitro*. **(A)** Wild-type (WT) or AIMp1 KO bone marrow-derived dendritic cell (BMDC) were matured for 48 h with cytokine cocktail, then cocultured with naive WT CD3^+^T-cells at a 1:10 ratio. IFN-γ from 3-day coculture supernatants was measured by ELISA (*n* = 7, biological repeats). **(B)** WT or AIMp1 KO BMDC were loaded with ovalbumin (OVA) antigen and matured for 48 h with cytokine cocktail, then cocultured with OT-II T-cells at a 1:20 ratio. IFN-γ from 3-day coculture supernatants was measured by ELISA (*n* = 8, biological repeats). **(C)** WT T-cells were harvested and lysed for Western blotting analysis of pSTAT1(Y701)/STAT1, pSTAT4(Y693), T-bet, and β-actin after 6 h of coculture with BMDC as in **(A)** (Representative of three independent experiments). **(D)** WT or AIMp1 KO BMDC were treated with LPS, and levels of proinflammatory cytokines IL-12p70, IL-6, and IL-1β were measured by ELISA (*n* = 7, technical repeats, pooled from two independent experiments). **(E)** WT or AIMp1 KO BMDC were treated with maturation cocktail or LPS and levels of CD86, CD40, and MHC II (I-A^b^) were measured by flow cytometry (normalized and pooled from multiple independent experiments). **(F)** Conditioned supernatant from WT or AIMp1 KO BMDC loaded with SIINFEKL + OVA antigen and matured for 48 h were mixed 1:1 with fresh media and no additional cytokines and used to treat fresh BMDC; Levels of *il12a, il12b*, and *il6* transcripts were measured by RT-PCR after 2 days (*n* = 3, biological repeats). Data are displayed as mean ± SEM. *p < 0.05, **p < 0.01, ***p < 0.001 as determined by two-way analysis of variance with Bonferroni *post hoc* test for multiple comparisons for **(B, D–F)**; two-tailed Student’s *t*-test for **(A)**.

Previous literature has suggested a potential role for AIMp1 in T_H_1 immunity (16–22, 45–47), and our work demonstrated BMDC-expressed AIMp1 is required for vaccine-mediated melanoma tumor rejection. To understand in greater detail whether BMDC-expressed AIMp1 can directly promote type 1 T-cell polarization, we began by investigating the function of AIMp1 in DC and T-cell crosstalk *in vitro*. Matured WT and AIMp1^−/−^ BMDC were cocultured with purified T-cells to test if the general activation of T-cells was affected by AIMp1 deficiency. WT CD3^+^T-cells cocultured with AIMp1^−/−^ BMDC for 72 h produced significantly less IFN-γ than ones cocultured with WT BMDC (Figure 2A). Additionally, OT-II T-cells which are transgenic CD4^+^T-cells specific for OVA antigen also produced significantly less IFN-γ when activated by OVA antigen-loaded AIMp1^−/−^ BMDC (Figure 2B), indicating antigen-specificity of the response. The production of IFN-γ from T-cells is a hallmark of T_H_1 polarization and is regulated by the intracellular signaling and transcriptional activities of STAT1, STAT4, and T-bet. Western blot analysis revealed significant reductions in STAT1 (Y701) and STAT4 (Y693) phosphorylation along with concomitant impaired upregulation of T-bet expression among T-cells cocultured for 6 h with AIMp1^−/−^ BMDC (Figure 2C). Given that WT and AIMp1^−/−^ mice exhibited no discernable differences in lymphocyte subset numbers within secondary lymphoid organs (Figure S3A in Supplementary Material) and that *in vitro* APC-free T-cell polarization assays indicated no *a priori* T_H_1 polarization deficit among AIMp1^−/−^ CD4^+^ T-cells (Figures S3B,C in Supplementary Material), the data indicated a direct role for BMDC-expressed AIMp1 in the observed T_H_1 polarization defect imparted during DC/T-cell interaction.

In addition to the presentation of antigen, DCs also express cytokines and costimulatory molecules necessary for T-helper cell polarization. Secretion of the master T_H_1-polarizing cytokine IL-12p70 (48) was dramatically impaired following LPS treatment of AIMp1^−/−^ BMDC in comparison to that secreted from WT BMDC, yet no deficits in other proinflammatory cytokines including IL-6 and IL-1β were observed (Figure 2D). Matured AIMp1^−/−^ BMDC also expressed lower levels of costimulatory molecules CD86 and CD40 as well as I-A^b^ (MHC class II) (Figure 2E). Using conditioned BMDC supernatants, we further demonstrated that supernatants derived from WT but not AIMp1^−/−^ OVA/SIINFEKL-loaded BMDC could induce upregulation of *il12a* and *il12b* mRNA transcripts in WT BMDC whereas any differential induction of other proinflammatory mRNA transcripts like *il6* was not observed (Figure 2F). Interestingly, AIMp1^−/−^ recipient BMDC failed to upregulate *il12a* and *il12b* transcripts even in the presence of WT BMDC-conditioned supernatants. These results showed that BMDC-expressed AIMp1 can promote T_H_1-specific programming *via* the regulation of specific cytokines and costimulatory molecules.

### Impaired p38 MAPK Signaling in AIMp1 Deficient BMDC Is Associated with Reduced T_H_1 Polarization

**Figure 3:**
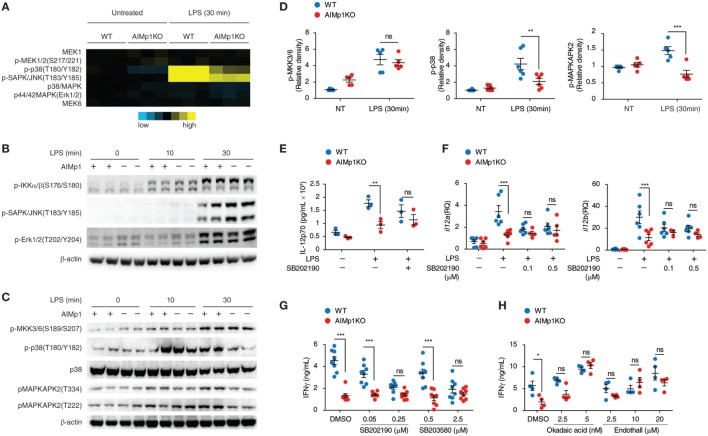
Bone marrow-derived dendritic cell (BMDC)-AIMp1 deficiency impairs MyD88 downstream p38 MAPK signaling. **(A)** Reverse phase protein array analysis of MAPK signaling pathway molecules in wild-type (WT) or AIMp1 KO BMDCs treated with LPS for 30 min; colored scale bar indicates normalized log2 expression levels of each protein over the WT untreated group (*n* = 3, biological repeats). **(B,C)** WT or AIMp1 KO BMDC were treated with LPS for 0, 10, or 30 min. Cells were harvested for Western blotting analysis of pIKKα/β and MAPK signaling pathway molecules (representative of three independent experiments). **(D)** Relative densitometry quantification of p-MKK3/6, p-p38, and p-MAPKAPK2 from multiple experiments (pooled from three independent experiments). **(E,F)** WT or AIMp1 KO BMDC were treated with LPS for 24 h in the presence of DMSO vehicle or 0.1 µM p38 MAPK inhibitor SB202190. Levels of IL-12p70 in the supernatant were measured by ELISA **(E)** (*n* = 3, technical repeats) and *il12a*/*il12b* transcripts were measured by RT-PCR **(F)** (*n* = 3–6, pooled from three independent experiments). **(G,H)** WT or AIMp1 KO BMDC were treated with maturation cytokine cocktail for 48 h in the presence of various concentrations (shown) of p38 inhibitors SB202190 and SB203580 **(G)** (*n* = 8, pooled from three independent experiments) and PP2A inhibitors Okadaic acid and Endothall **(H)** (*n* = 4, technical repeats, representative of two independent experiments); cells were then washed and cocultured with WT T-cells at a 1:10 ratio for 3 days; levels of IFN-γ in supernatants were measured by ELISA. Data are displayed as mean ± SEM. *p < 0.05, **p < 0.01, ***p < 0.001 as determined by two-way analysis of variance with Bonferroni *post hoc* test for multiple comparisons.

The observation that AIMp1 in BMDC specifically promotes downstream T_H_1 T-cell polarization led us to investigate the underlying immune signaling pathways responsible. Previous work has demonstrated that the impact of AIMp1 on cell signaling can be complex, multifaceted, and frequently cell-type specific. Recombinant AIMp1 was reported to induce phosphorylation of ERK1/2, p38 MAPK, and c-Jun and to be critical for the production of TNF-α in THP-1 cells (16, 49). Activation of these MAP kinase signaling pathways was also induced by recombinant AIMp1 in skin fibroblasts; however, only ERK1/2 phosphorylation was responsible for AIMp1-mediated wound repair (15). Conversely, IL-12 production in primary macrophages induced by recombinant AIMp1 was reported to involve both NF-κB and p38 MAPK (22). To determine which of these various signaling pathways could be involved in BMDC-expressed AIMp1 signaling, we performed RPPA analysis of over 200 signaling proteins among WT and AIMp1^−/−^ BMDC at 30 min post-LPS treatment. This comprehensive analysis implicated significant impairment of early p38 MAPK signaling in AIMp1^−/−^ BMDC but no observed deficits in ERK, JNK, nor NF-κB signaling (Figure 3A). By Western blot analysis, we confirmed that AIMp1 deficiency did not impact the activation of NF-κB signaling in response to LPS treatment as measured by IKK phosphorylation (Figure 3B) and IκB degradation (data not shown). Similarly, there were no observed differences in phosphorylation levels of ERK1/2 or SAPK/JNK MAPK (Figure 3B). In contrast, phosphorylation of p38 MAPK and downstream substrate MAPKAPK2 were significantly impaired in AIMp1^−/−^ BMDC, whereas activation of MKK3/6 directly upstream of p38 MAPK was not (Figures 3C,D), suggesting that AIMp1 might exert its regulatory effects specifically at the level of p38 MAPK.

The p38 MAPK signaling pathway within BMDC has previously been linked to inflammatory function and induction of T_H_1 polarization (50, 51). Treatment with p38 MAPK small molecule inhibitors (SB202190 and SB203580) led to downregulated phosphorylation of the p38 MAPK downstream substrate MAPKAPK2 (Figures S4A–D in Supplementary Material). In the presence of p38 MAPK inhibitors, IL-12 protein and mRNA transcripts were also downregulated in LPS-treated WT BMDC to levels comparable to those of AIMp1^−/−^ BMDC (SB202190 shown Figures 3E,F). Furthermore, T-cells cocultured with p38-inhibited WT BMDC produced IFN-γ at levels similar to those cocultured with AIMp1^−/−^ BMDC in a dose-dependent fashion (Figure 3G). All inhibitor-treated BMDC were thoroughly washed prior to T-cell coculture, mitigating any potential effects of the inhibitors on the T-cells themselves. Further investigation will be required to explain how specific impairment of p38 MAPK mediates downregulation of BMDC IL-12 production and IFN-γ from cocultured T-cells. Nonetheless, these results demonstrated that p38 MAPK inhibition in WT BMDC mimics the AIMp1^−/−^ phenotype with regard to T_H_1 polarization.

p38 MAPK signaling is regulated by both kinases and phosphatases. PP2A and DUSP1/MKP1 are among several key regulatory phosphatases known to control the activity of p38 MAPK (52, 53). While there were no observed differences in levels of DUSP1 nor regulatory subunits A and B of PP2A following LPS treatment, levels of PP2A catalytic subunit C were significantly elevated by 60% among AIMp1^−/−^ BMDC (Figure S5 in Supplementary Material). Direct binding of AIMp1 to PP2A was also suggested by mass spectrometry analysis of proteins that coimmunoprecipitated with AIMp1 (data not shown). Therefore, we used small molecule inhibitors of DUSP1 and PP2A on AIMp1^−/−^ BMDC to determine if either of these could rescue T_H_1 responses. While inhibition of DUSP1 had no impact on T_H_1 impairment (data not shown), inhibition of PP2A in AIMp1^−/−^ BMDC was sufficient to restore p38 MAPK phosphorylation (Figures S4E–H in Supplementary Material) and downstream IFN-γ secretion of cocultured lymphocytes to WT levels (Figure 3H). These results suggested that upregulated PP2A phosphatase activity in AIMp1^−/−^ BMDC could account for decreased p38 MAPK function, though delineation of the complete mechanism may require additional investigation. Nonetheless, the data indicated that impaired p38 MAPK signaling is at least partially responsible for the reduction of downstream T_H_1 polarization in AIMp1^−/−^ BMDC.

### BMDC-Intrinsic AIMp1 Impacts Transcriptional Regulation of Immune Responses

**Figure 4:**
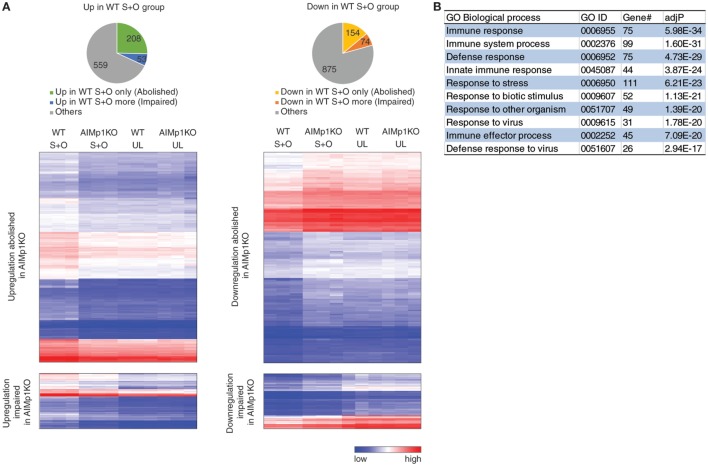
Bone marrow-derived dendritic cell (BMDC)-AIMp1 influences transcriptional regulation of immune responses. Wild-type (WT) or AIMp1 KO BMDC were treated with maturation cytokine cocktail for 48 h. UL: unloaded; S + O: loaded with SIINKFEL and OVA (*n* = 3, biological repeats). **(A)** Pie charts of 820 upregulated (left, “up in WT S + O group”) and 1,103 downregulated (right, “down in WT S + O group”) genes in WT S + O group over WT UL group among 26,336 total coding genes as determined by mouse transcriptional array of WT BMDC. Charts and hierarchical heatmaps indicate the 261 upregulated genes (left) and 228 downregulated genes (right), the differential regulation of which was either abolished or significantly impaired by the absence of dendritic cell AIMp1. **(B)** List of Gene Ontology (GO) biological processes analyzed by WEBGESTALT of all differentially regulated genes (“abolished” and “impaired”) plotted in **(A)**.

To study the global effects of AIMp1 deficiency on DC immune responses, we compared the transcriptional profile of WT and AIMp1^−/−^ BMDC, both mock loaded and antigen loaded, using the Mouse Exon 1.0 ST Array, comprised of over 1.2 million unique probe sets that correspond to 554,000 unique ESTs. This analysis identified 1,923 protein-encoding genes differentially expressed in a statistically significant fashion. Of 820 upregulated genes, the absence of AIMp1 impaired or abolished upregulation of 261; and of 1,103 downregulated genes, the absence of AIMp1 impaired or abolished downregulation of 228 (Figure 4A). Identification and *in silico* analysis of the 489 genes dependent upon AIMp1 expression suggested prominent AIMp1 regulation in a variety of immune processes, including the innate immune response and antiviral defense (Figure 4B). The top 50 most differentially regulated genes are shown in Table 1. Although not among the top 50, genes from the mouse major histocompatibility complex H-2 gene cluster which encodes proteins associated with antigen presentation were also identified. Important antiviral genes including interferon-activated genes (*Ifit1, Ifit2, Ifit3*, etc.) or interferon regulatory factor genes (*Irf7*), as well as innate immune sensors for viral RNA such as *Oas* family genes and *Ddx58*, were also shown to be critically regulated. The upregulation of such antiviral programming is known to be closely related to the antitumor potential of DCs (54), explaining the inability of AIMp1^−/−^ BMDC vaccines to mediate the rejection of B16-OVA tumors. The analysis also identified 124 differentially regulated miRNAs and hundreds of long non-coding (lnc) RNAs (data not shown), the biological significance of which may be delineated in future studies.

**Table 1:** Top 50 genes differentially regulated by AIMp1 in response to antigen loading.

Gene symbol	Gene name	Regulation by AIMp1	Fold change (WT vs. AIMp1KO S + O)	Analysis of variance *p*-value	Function
*Ifit1*	Interferon-induced protein with tetratricopeptide repeats 1	Up	20.55	0.000103	Innate antiviral response; IFN-induced
*Cxcl1*	Chemokine (C–X–C motif) ligand 1	Up	11.46	0.000003	Neutrophil ohemotaxis
*Ch25h*	Cholesterol 25-hydroxylase	Down	10.33	0.000139	Lipid metabolic process
*Rsad2*	Radical S-adenosyl methionine domain containing 2	Up	9.75	0.000303	Innate antiviral response; IFN-induced
*Il1f6*	Interleukin 1 family, member 6	Up	9.56	0.000035	Induction of dendritic cell maturation and inflammation
*Car4*	Carbonic anhydrase 4	Down	7.4	4.40E−07	Metabolic process
*Mx2*	Myxovirus (Influenza Virus) Resistance 2	Up	7.24	0.000292	Innate antiviral response
*Oasl1*	2-5 oligoadenylate synthetase-like 1	Up	7.09	0.000802	Innate antiviral response
*Irf7*	Interferon regulatory factor 7	Up	7.05	0.000106	Innate antiviral response; IFN-induced
*Spp1*	Secreted phosphoprotein 1	Down	6.8	0.00006	Negative regulation of apoptotic process
*Ptgs2*	Prostaglandin-endoperoxide synthase 2	Up	6.08	0.00001	Production of inflammatory prostaglandins
*Mmp19*	Matrix metallopeptidase 19	Down	5.99	0.000331	Collagen catabolic process
*Hdc*	Histidine decarboxylase	Up	5.96	0.000094	Histamine metabolic process
*Mgl2*	Macrophage galactose N-acetyl-galactosamine specific lectin 2	Down	5.36	0.000101	Glycosylated antigens uptake
*Lyz1*	Lysozyme 1	Down	5.22	0.000034	Defense response to bacterium
*Gm15056*	Predicted gene 15056	Up	5.15	0.000272	Predicted defensin beta 52
*Ifi204*	Interferon activated gene 204	Up	5.02	0.000054	Type I IFN response; intrinsic apoptotic signaling pathway
*Ptges*	Prostaglandin E synthase	Up	4.97	0.000111	Prostaglandin biosynthetic process
*Mnda*	Myeloid cell nuclear differentiation antigen	Up	4.58	0.000032	Granulocyte–monocyte lineage cell
*AI607873*	Expressed sequence AI607873	Up	4.57	0.000066	Novel interferon-activable protein
*Ifi202b*	Interferon activated gene 202B	Up	4.54	0.000114	Regulation of transcription
*Xaf1*	XIAP-associated factor 1	Up	4.53	0.000216	Apoptotic process
*Oasl2*	2-5 oligoadenylate synthetase-like 2	Up	4.48	0.000337	Innate antiviral response; IFN-induced
*Prss34*	Protease, serine 34	Down	4.43	0.000624	Proteolysis
*Trim30c*	Tripartite motif-containing 30C	Up	4.39	0.000119	Regulation of transcription
*Ddx60*	DEAD (Asp-Glu-Ala-Asp) box polypeptide 60	Up	4.26	0.000024	Innate antiviral response
*Ms4a3*	Membrane-spanning 4-domains, subfamily A, member 3	Down	4.1	0.000071	Regulation of cell cycle
*Ccl7*	Chemokine (C–C motif) ligand 7	Up	4.09	0.003074	Monocyte chemotaxis
*Pla1a*	Phospholipase A1 member A	Down	3.99	0.000011	Lipid metabolic process
*Pyhin1*	Pyrin and HIN domain family, member 1	Up	3.93	0.000229	Tumor suppressor
*Tbxas1*	Thromboxane A synthase 1, platelet	Down	3.88	0.000093	Prostaglandin biosynthetic process
*Hamp*	Hepcidin antimicrobial peptide	Up	3.86	0.000221	Defense response to bacterium
*BC094916*	cDNA sequence BC094916	Up	3.78	0.000656	Regulation of transcription
*Serpinb2*	Serine (or cysteine) peptidase inhibitor, clade B, member 2	Up	3.75	0.000379	Negative regulation of apoptotic process
*Axl*	AXL receptor tyrosine kinase	Down	3.72	0.000185	Inhibition of Toll-like receptor (TLR) signaling
*Egr2*	Early growth response 2	Down	3.72	0.001619	Regulation of transcription
*Enpp2*	Ectonucleotide pyrophosphatase/phosphodiesterase 2	Down	3.65	0.000082	Lipid metabolic process
*Trim30d*	Tripartite motif-containing 30D	Up	3.63	0.000141	Protein binding
*Oas2*	2-5 oligoadenylate synthetase 2	Up	3.59	0.000214	Innate antiviral response; IFN-induced
*Hamp2*	Hepcidin antimicrobial peptide 2	Up	3.58	0.000127	Defense response to bacterium
*Casp4*	Caspase 4, apoptosis-related cysteine peptidase	Up	3.55	0.000034	Apoptotic process
*Clec10a*	C-type lectin domain family 10, member A	Down	3.54	0.000062	Cell adhesion; immune regulation
*Orm1*	Orosomucoid 1	Up	3.5	0.00191	Protein transport; acute-phase immune response
*Fam115c*	Family with sequence similarity 115, member C	Down	3.45	0.00171	Cation channel TRPM8 regulation; hematopoietic progenitor cell differentiation
*Usp18*	Ubiquitin specific peptidase 18	Up	3.44	0.000714	Proteolysis of antiviral molecule ISG15
*Susd2*	Sushi domain containing 2	Up	3.42	0.002498	Potential tumor suppressor
*Fgr*	Gardner-Rasheed feline sarcoma viral (Fgr) oncogene homolog	Down	3.42	0.000233	Integrin-mediated signaling pathway
*Oas3*	2-5 oligoadenylate synthetase 3	Up	3.41	0.000626	Innate antiviral response; IFN-induced
*Mertk*	c-mer proto-oncogene tyrosine kinase	Down	3.38	0.000764	Cell–cell signaling; inhibition of TLR signaling
*Clec4b2*	C-type lectin domain family 4, member b2	Down	3.35	0.000514	Antigen processing and presentation

### AIMp1 Deficiency in Mice Impairs Innate and Adaptive Antiviral Immunity to Influenza Virus Infection

**Figure 5:**
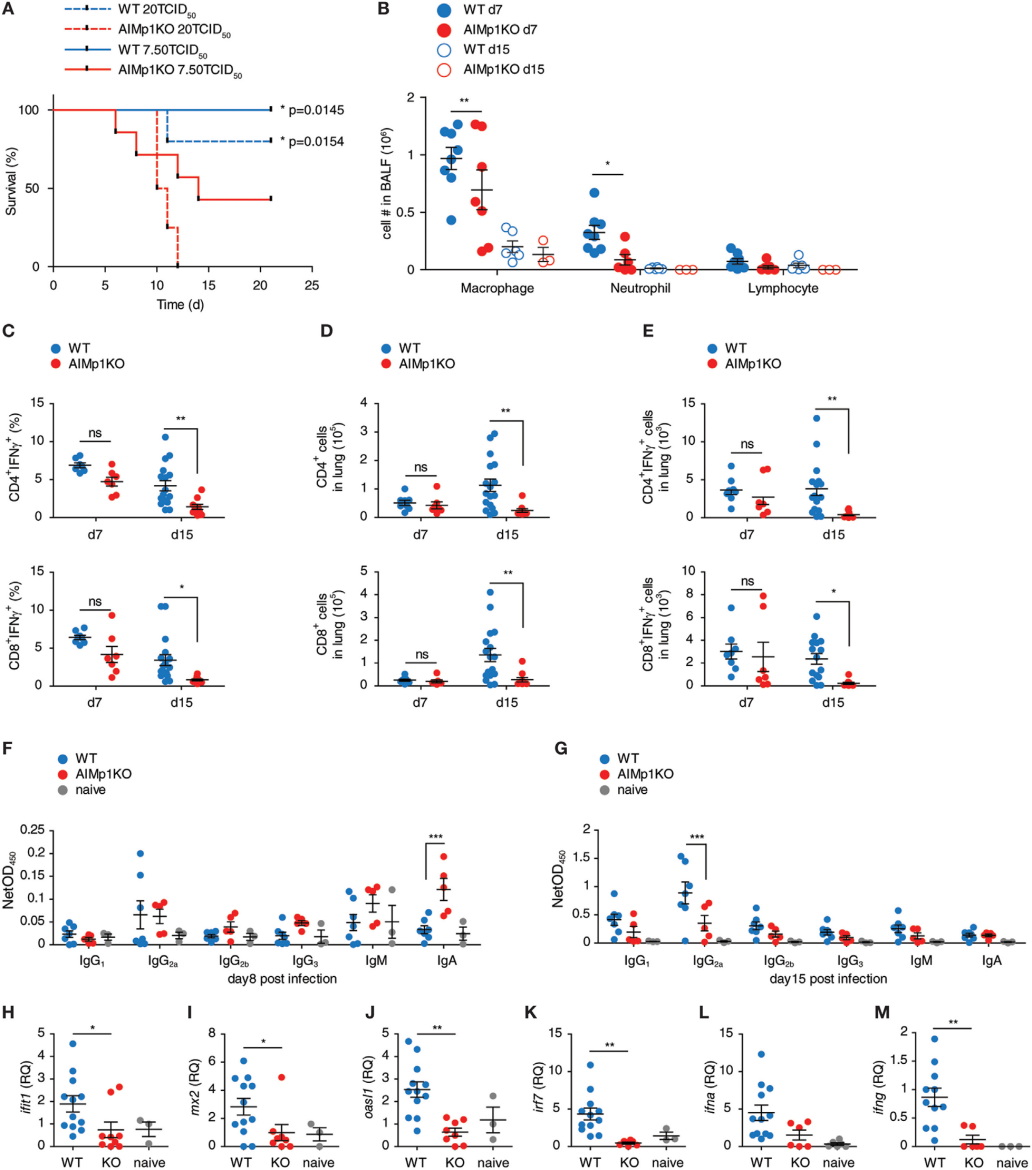
AIMp1-deficient mice exhibit defective antiviral immunity. **(A)** Kaplan–Meier survival analysis of wild-type (WT) or AIMp1 KO animals challenged with aerosolized A/HongKong/8/68 (H3N2) (A/HK/68) mouse lung adapted strain of influenza A virus. *p < 0.05 was determined by log rank (Mantel-Cox) test between WT and AIMp1 KO mice infected with corresponding dose (*n* = 5–8). **(B)** Bronchoalveolar lavage fluid analysis of mice infected with an estimated 6.25 TCID_50_ per mouse of A/HK/68 on postinfection days 7 and 15 (*n* = 3–8). **(C–E)** Intracellular flow cytometry analysis of lung lymphocytes from mice infected with an estimated 6.25 TCID_50_ of A/HK/68 as in **(B)** on postinfection days 7 and 15 (*n* = 8–17, pooled data from two independent experiments). **(C)** IFN-γ^+^CD4^+^ and CD8^+^ as a percent of total CD4^+^ and CD8^+^. **(D)** Total numbers of CD4^+^ and CD8^+^ cells. **(E)** Total numbers of IFN-γ^+^ CD4^+^ and CD8^+^ cells. **(F,G)** Serum from mice infected as in **(B)** were analyzed for anti-HA specific antibody isotypes IgG_1_, IgG_2a_, IgG_2b_, IgG_3_, IgM, and IgA on postinfection day 8 **(F)** and day 15 **(G)** (*n* = 3–7, representative of two independent experiments). **(H–M)** Cells in BALF from mice infected as in **(G)** were analyzed for select antiviral and interferon-responsive gene expression. **(H)** *ifit1*, **(I)** *mx2*, **(J)** *oasl1*, **(K)** *irf7*, **(L)** *ifna1*, **(M)** *ifng*. [**(H–M)**: *n* = 3–12, pooled data from two independent experiments]. Data are displayed as mean ± SEM. *p < 0.05, **p < 0.01, ***p < 0.001 as determined by two-way ANOVA in **(B–G)** and one-way ANOVA in **(H–M)** with Bonferroni *post hoc* test for multiple comparisons.

We have demonstrated the importance of AIMp1 in T_H_1 polarization during antitumor immunity both *in vivo* and *in vitro*; however, the microarray data indicated that AIMp1 function might also be highly relevant to immunity against intracellular virus infection. Moreover, recent studies have indicated that AIMp1 is physiologically upregulated in bronchial epithelial cells in response to influenza virus infection (18). To study the function of AIMp1 in antiviral immunity, we challenged WT and AIMp1^−/−^ mice with varying titers of aerosolized A/Hong Kong/8/68 (H3N2) influenza A virus. A high infectious dose of 20 TCID_50_ per mouse was sufficient to cause 100% lethality among AIMp1^−/−^ mice yet only resulted in 20% lethality among WT mice (Figure 5A). A sub-lethal dose of 7.50 TCID_50_ per mouse still caused 60% mortality among AIMp1^−/−^ mice versus 0% among WT animals (Figure 5A). Survivors in both cohorts exhibited similar levels of weight loss and peak virus titers (data not shown) following the sub-lethal dose. Despite no overt clinical phenotype at the earliest stages of infection amongst the AIMp1^−/−^ survivors, AIMp1 deficiency mediated significant effects on innate and adaptive immune responses on days 7 and 15 postinfection. On day 7, we observed elevated levels of infiltrating neutrophils and macrophages in BALF derived from WT mice whereas these were significantly reduced in BALF derived from AIMp1^−/−^ mice at this time point (Figure 5B). On day 7, there were similar levels of lung-infiltrating IFN-γ^+^ T-cells observed in both WT and AIMp1^−/−^ mice; however, by day 15, IFN-γ^+^ lung-infiltrating T-cells were substantially reduced in AIMp1^−/−^ mice (Figures 5C–E). Analysis of anti-HA isotype-specific antibody titers in serum collected from mice on days 8 and 15 postinfection demonstrated that IgG_2a_, the T_H_1-specific antibody isotype in the mouse (55), was the predominant anti-HA isotype produced in WT mice at day 15 postinfection. In contrast, elevation of this isotype was highly impaired in AIMp1^−/−^ animals (Figures 5F,G). No statistical differences were observed among any other anti-HA antibody isotype. We performed real-time RT-PCR on BALF samples to validate the *in vivo* expression of selected differentially expressed antiviral genes identified by the microarray analysis above. *Ifit1, mx2, oasl1, irf7, ifna1*, and *ifng* were significantly upregulated following infection in WT mice but not in AIMp1^−/−^ mice (Figures 5H–M). Collectively, these results suggested that AIMp1 deficiency leads to insufficient innate and adaptive antiviral immune responses, correlating with the higher levels of mortality observed in these animals.

### Primary Tumor AIMp1 Expression Is Associated with a Significant Survival Advantage in Human Cancer

**Figure 6:**
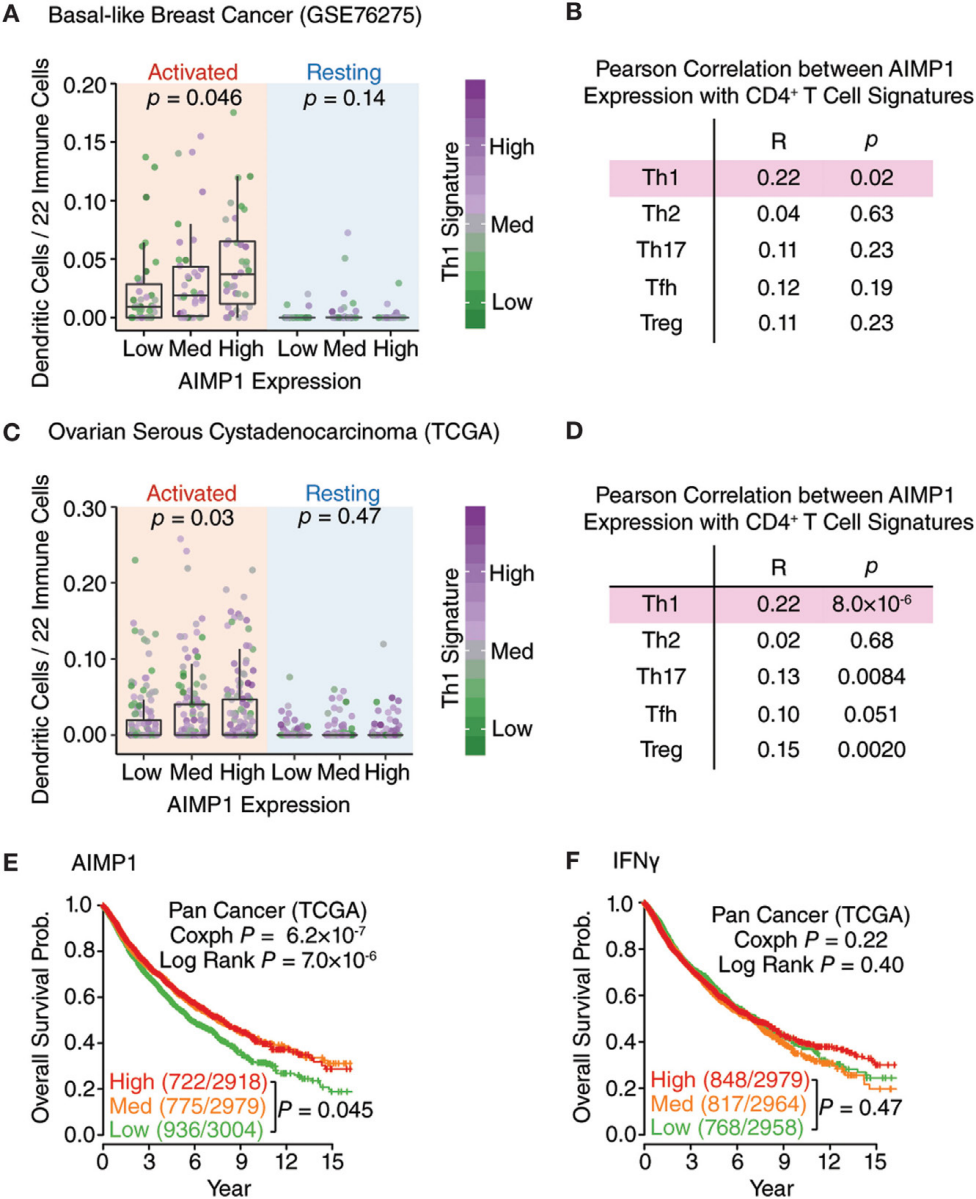
AIMp1 expression in primary human tumors is correlated with T_H_1 immunity and enhanced survival. **(A,C)** Box–Whisker plots showing the relative frequency of activated/resting dendritic cells in the different groups of basal-like breast cancer (*n* = 265, GSE76275) **(A)** and ovarian cancer (*n* = 419, TCGA) **(C)** classified by the AIMP1 expression. The T_H_1 signature was indicated by the color side bar. Each point represents one tumor sample. The relative percentages of dendritic cells were predicted using CIBERSORT. **(B,D)** Correlation of different T-cell subsets with AIMP1 expression. The different T-cell subsets signatures were predefined and described in methods. For the Box–Whisker plots, the upper and lower hinges correspond to the first and third quartiles, and the upper and lower whiskers are highest and lowest values that are within 1.5 × IQR (interquartile range) of the hinge. *P* values were calculated using two-tailed analysis of variance (ANOVA) **(A,C)** and Student’s *t*-test on the Pearson correlation coefficient **(B,D)**. **(E,F)** Kaplan–Meier plots of the probability of overall survival across TCGA pan-cancer data set (*n* = 8,901) divided into tertiles based on the expression of AIMP1 and IFN-γ, respectively. The numbers of non-surviving and total patients in each group are indicated in the parentheses. The *p*-value was calculated by cox regression and log rank test.

After decades of retrospective study, immune response signatures are now utilized as prospective prognostic indicators of patient outcome in cancer. Recently, analysis of 297 glioma samples from the Chinese Glioma Genome Atlas for local immune signature genes identified *AIMP1, FOXO3*, and *ZBTB16* as positive prognostic indicators well correlated with patient overall survival (OS) (20). Based upon this observation, we sought to expand further upon the relationship between tumor-associated AIMp1 and good outcomes in cancer. First, we utilized CIBERSORT (30, 31) with the previously validated GSE76275 basal-like breast cancer and TGCA serious ovarian cystadenocarcinoma databases to examine the relationship between the relative percentage of 22 different immune cell subset signatures and AIMp1 expression levels in primary basal-like breast and ovarian tumors. In basal-like breast cancer, elevated levels of *AIMP1* expression were found to be positively correlated with both increased numbers of activated tumor-infiltrating DCs and a T_H_1 T-cell signature (Figure 6A). There were no significant correlations between *AIMP1* expression and any other T_H_ signature including T_H_2, T_H_17, T_FH_, and T_REG_ (Figure 6B). A similar observation was made using the TCGA ovarian cancer dataset (Figures 6C,D). While these data could not definitively identify DCs as the primary source of *AIMP1* expression within primary tumors, they were highly suggestive and nonetheless affirmed the impactful relationship between AIMp1 and T_H_1 immunity in a relevant, real world setting of human disease. To better correlate *AIMP1* expression with outcomes data, patients in individual tumor databases were stratified into high, medium, and low tertiles by *AIMP1* expression level, and Kaplan–Meier survival analysis was performed. Strong trends toward enhanced survival among patients with high *AIMP1* expression were observed in ovarian cancer, breast cancer (data not shown) and melanoma. IFN-γ expression was shown to be critically important to survival in melanoma as reported previously (Figure S6 in Supplementary Material). A similar survival analysis was then performed using all 8,901 patient samples in the pan-cancer TCGA database for which outcomes data could be determined. This large-scale analysis demonstrated a highly significant 70% survival advantage among patients in medium or high level *AIMP1* expression tertiles at 15 years postdiagnosis (Figure 6E). Interestingly, IFN-γ gene expression in these tumors correlated with only a non-significant 20% survival advantage among patients in the highest tertile in comparison to those in the medium and low tertiles (Figure 6F). Hence for tumors already thought to be immunogenic (i.e., melanoma), the data validated the importance of IFN-γ expression to survival. However, for the sum total of all tumors, the data indicated that primary tumor AIMp1 expression was at least as important as that of IFN-γ to long-term survival in cancer.

## Discussion

Cell-mediated immune responses by CD4^+^ T helper 1 (T_H_1) cells and CD8^+^ CTLs are critical for the clearance of transformed or infected host cells. This process is largely dependent upon effective antigen presentation, specific cytokine secretion, and polarized costimulatory molecule expression by DCs. Here we found that BMDC-expressed AIMp1 promotes T_H_1 polarization during antitumor immune responses and host AIMp1 expression is critical for antiviral defense. A structural component of the multienzyme aminoacyl-tRNA synthetase (mARS) complex, AIMp1 was identified as the cytosolic precursor of the inflammatory cytokine endothelial monocyte-activating polypeptide-II (EMAPII) (56, 57). More recently, AIMp1 was reported to be secreted by cancer cells under conditions of stress in a manner substantially different from that of EMAPII (17). While the function of the enigmatic mARS complex remains largely uncharacterized, recent data suggest a role in both detection and defense of viral infection (18). Because recombinant human AIMp1 protein can activate pro-inflammatory programs in multiple cell types including monocytes, macrophages, DCs, B-cells, and others (16, 21, 22), it has been hypothesized that dissociation of AIMp1 from the mARS complex following inflammatory stimuli might permits a functionally distinct role of the soluble protein as a novel pro-inflammatory cytokine (58). *In vivo*, recombinant human AIMp1 can inhibit the growth of multiple tumor cell lines or transplanted tumors (38, 40), possibly by upregulation of tumor necrosis factor receptor and also through negative regulation of the immunosuppressive microenvironment (59). Genetic ablation of AIMp1 has also been linked to T_H_2-biased airway hyperreactivity in a model of allergic airway inflammation (19). Despite these observations, there has been no direct *in vivo* evidence that AIMp1 functions to promote T_H_1 immune processes during antitumor or antiviral defenses. The cell type-specific role of AIMp1 in T_H_1 immunity remains poorly understood, and we are the first to investigate its function within DCs in the context of host immunity against cancer and intracellular infection. Our study is also the first attempt to explain the molecular mechanism of its immunoregulatory functions.

We observed that the absence of AIMp1 in BMDC leads to lower levels of costimulatory and antigen presentation molecules as well as secretion of the T_H_1-polarizing cytokine IL-12 following treatment with maturation stimuli. The production of pro-inflammatory cytokines IL-6 and IL-1β was not affected. Conditioned supernatant experiments suggest that AIMp1 released from antigen-loaded BMDC could also be pro-inflammatory and T_H_1-polarizing. Subsequently, T-cells stimulated by AIMp1^−/−^ BMDC could upregulate IFN-γ production above baseline but to significantly lower levels than WT BMDC. These differences were observed in both generic T-cell activation without specific antigen and in OT-II T-cell activation by OVA. T_H_1 programming is preceded by the phosphorylation of STAT1 and STAT4 and induction of T-bet transcription factor at an early stage of T-cell activation. We observed significantly impaired induction of these molecules in WT T-cells activated by AIMp1^−/−^ BMDC. BMDC-expressed AIMp1 is critical for the clearance of immunogenic B16-OVA melanoma tumor by SIINFEKL + OVA-loaded BMDC vaccination *in vivo*; whereas the presence of AIMp1 in the germline of recipient mice appeared to have almost no impact upon tumor regression nor survival. These data provide direct evidence that BMDC-expressed AIMp1 functions as a T_H_1 polarizing effector molecule, potentially by upregulation of specific cytokines and costimulatory molecules that permit polarization of effector adaptive immune responses toward T_H_1.

While we established the critical role of DC-expressed AIMp1 in the induction of T_H_1 responses, potential contributions of AIMp1 in other immune cell subtypes to immune defects in AIMp1^−/−^ animals were not overtly obvious. Initial observations suggested few defects in AIMp1^−/−^ T-cells. The compositions of the CD3^+^CD4^+^ and CD3^+^CD8^+^ T-cell compartments, as well as CD19^+^ B-cells, and NK1.1^+^ NK cells, were similar between WT and AIMp1^−/−^ mice in secondary lymphoid organs, suggesting that an absence of AIMp1 does not impart a significant developmental defect. The ability of T_H_0 CD4^+^ cells to differentiate into IFN-γ-secreting T_H_1 cells in a DC-free T-cell differentiation assay was also not impacted by the absence of AIMp1. Even further, the ability to inhibit B16-OVA melanoma tumor growth through vaccination depended solely upon the AIMp1 phenotype of the BMDC vaccine but not upon any other host immune cell in the recipient mouse. Nonetheless, stimulation of AIMp1^−/−^ T-cells with WT DC could result in a partial IFN-γ secretion defect in some *in vitro* experiments (data not shown), even though such deficits were not observed *in vivo*. The role of AIMp1 in other immune cell subsets might be important in a model-dependent fashion and could be addressed using conditional knockout models and additional experimentation. Additionally, our study did not distinguish between cell intrinsic and cell extrinsic functions of AIMp1. In addition to DC-intrinsic effects, it is possible that AIMp1 released from BMDC exerts proinflammatory and T_H_1 polarizing activities on other immune cells, including T-cells, NK cells, B-cells, and MDSC. Further studies will be necessary to determine the mechanism for AIMp1 expression/upregulation under T_H_1-promoting conditions as well as whether AIMp1 functions differently in intracellular and extracellular environments.

Mechanistically, we found that AIMp1 promotes T_H_1 polarization at least partially through positive regulation of p38 MAPK in DC. Several classes of phosphatases play important roles in the negative regulation of p38 MAPK as well as other members of the MAPK family (60). Following an initial screen by mass spectroscopy that suggested direct binding between AIMp1 and the phosphatase PP2A, we identified higher levels of the PP2A catalytic subunit C among AIMp1^−/−^ BMDC following LPS treatment. Subsequently, we demonstrated that small molecule inhibition of PP2A in AIMp1^−/−^ BMDC could rescue the impaired T_H_1 phenotype of downstream effectors. Despite the fact that PP2A targets kinases other than p38 MAPK, the data support a hypothesis whereby AIMp1 promotes activation of p38 MAPK by preventing PP2A-mediated dephosphorylation. The data also do not preclude the possibility that AIMp1 promotes phosphorylation of p38 MAPK *via* other regulatory kinases. Meanwhile, small molecule inhibition of p38 MAPK downregulated the production of IL-12 from WT BMDC and IFN-γ from cocultured T-cells in a dose-dependent fashion while having little or no effect on AIMp1^−/−^ BMDC. The data indicate that a degree of p38 MAPK pathway activation in BMDC is critical for downstream T_H_1 polarization (61), which remains at a low level in the absence of AIMp1. Activation of p38 MAPK is responsible for the downstream regulation of multiple inflammatory responses, the impairment of which cannot fully explain the specific effect of AIMp1 on DC function. Importantly, apart from MyD88, LPS stimuli can also activate TRIF dependent IRF3/7 signaling pathways, and microarray analysis indicated differential regulation of *irf7* in the absence of AIMp1. Further, p38 MAPK drives phosphorylation and dimerization of Fos and Jun monomers to generate the AP-1 transcription factor, and AP-1 promoter specificity is dependent upon the complex combinatorial patterns of the monomeric subunits (62, 63). Consistent with this paradigm, genetic ablation of c-fos dysregulates IL-12 production (64), whereas c-jun phosphorylation (65) and homodimerization (66) suppresses T_H_1 responses and enhance IL-1β secretion. Because we observed a significant reduction in c-fos phosphorylation by RPPA analysis in AIMp1^−/−^ DC (data not shown) as well as upregulated IL-1β secretion, future studies may focus on whether and how AIMp1 governance of AP-1 specificity contributes to immune regulation and T_H_ polarization. A potential role for IRF7 signaling will be investigated as well.

We have shown AIMp1 is also critical for antiviral host defense in a model of influenza virus infection. The lack of effective innate and adaptive antiviral immunity observed among AIMp1^−/−^ mice following sublethal infection may be responsible for the higher mortality observed among these animals following fully lethal doses. AIMp1 deficiency resulted in lower levels of local inflammation, fewer T_H_1 effector T-cells, and lower levels of T_H_1 isotype-specific antibodies. Neutrophils that respond quickly to infection infiltrated the lung at a significantly lower level in AIMp1^−/−^ animals at day 7 postinfection. While only subtle differences in CD4^+^IFN-γ^+^ and CD8^+^IFN-γ^+^ lung T-cells were observed at the early time point, these important effectors were practically absent in AIMp1^−/−^ animals by day 15 postinfection. Immune cells in BALF expressed much lower levels of selected antiviral genes in AIMp1^−/−^ animals in accordance with the microarray analysis of *in vitro*-derived WT and AIMp1^−/−^ BMDC. The major BALF immune cell component is resident macrophages, indicating the presence of AIMp1 within innate APC could play an important role in regulating antiviral gene expression profiles. This result remains consistent with microarray analysis of WT and AIMp1^−/−^ BMDC. Although the precise functions of AIMp1 in DC remain to be delineated in the context of influenza virus infection, its role in the transcriptional regulation of antiviral immune genes could partially explain the observed results *in vivo*. As discussed previously, AIMp1 release can be induced by cell stress (15–17). The potential synergy of cell stress and/or other factors with better-characterized signals such as innate pattern recognition toward the generation of physiologic immune responses was not addressed here experimentally but should be thoughtfully considered. The data suggested that AIMp1 also impacts B-cell or Tfh cell function with regard to Ig isotype of antibodies critical for influenza virus protection. Thus, further studies need to be carried out using conditional knockout mice to understand the physiologic functions of AIMp1 in this infectious model system.

Elevated expression of *AIMP1* was recently shown to correlate positively with OS in glioblastoma (20). Given this important observation and the clear association of AIMp1 with T_H_1 immune processes, we analyzed the relationship between *AIMP1* expression and patient outcome among the nearly 9,000 primary tumor samples in the pan-cancer TCGA database that could be linked to outcomes data. These data demonstrated a remarkable 70% survival advantage at 15 years postdiagnosis among patients with high and medium levels of *AIMP1* expression. A similar trend was observed among individual cancer types but was only statistically significant in the melanoma database, presumably due to sampling size effects and other disease-specific factors including stage at diagnosis, treatment regimen, and general survival characteristics. Nonetheless, the data suggest that AIMp1 plays a genuine and significant role in the generation of durable antitumor immunity. We also demonstrated that *AIMP1* expression could be correlated with the immune signatures of activated tumor-infiltrating DC and T_H_1 T-cell subsets. The data showed a correlation between human *AIMP1* expression, DC T_H_1 polarization, and patient survival. While prospective genetic studies will be needed to understand the source of AIMp1 expression within the tumor tissues, the results serve to expand the understanding of AIMp1 functionality from *in vitro* experiments and animal models to human study.

In summary, the present study demonstrates that DCs utilize AIMp1 to initiate T_H_1 polarization. Genetic ablation of AIMp1 reduced the ability of DC to express IL-12 and other costimulatory signals, significantly impairing the generation of downstream effector T_H_1 responses. This process is partially facilitated by p38 MAPK signaling and downstream transcriptional regulation. Experimentally, AIMp1 was shown to be critical in antiviral and antitumor immunity, and analyses of TCGA databases indicated that AIMp1 expression in primary tumors is highly correlated with long-term survival. Additional studies will be required to determine the precise molecular signals that impact physiologic AIMp1 release as well as how such signals synergize with innate pattern recognition to affect durable cell-mediated immunity.

## Ethics Statement

Experiments were performed utilizing NIH and United States Department of Agriculture guidelines, The Public Health Service Policy on Humane Care and Use of Laboratory Animals, and experimental protocols approved by the Baylor College of Medicine Investigational Animal Care and Use Committee (IACUC Protocol numbers AN-1428 and AN-2307).

## Author Contributions

DL, LT, SH, BG, JL, and WD designed the research studies. DL, LT, RY, YB, and FJ conducted the experiments. DL, LT, RY, YB, FJ, and SH acquired the data. DL, LT, MH, VK, SP, FJ, SH, XZ, FK, DC, BG, JL, and WD analyzed the data. DK, SK, SH, XZ, FK, DC, BG, JL, and WD provided reagents. DL, MH, VK, SP, FK, DC, BG, JL, and WD wrote the manuscript.

## Conflict of Interest Statement

The authors declare that the research was conducted in the absence of any commercial or financial relationships that could be construed as a potential conflict of interest.

## Supplementary Material

Figure S1 AIMp1 within BMDC promotes control of B16F10 melanoma. Wild-type (WT) C57BL/6:129 F1 animals were challenged with 50,000 B16F10 melanoma tumor cells s.c. on day 0. On day 7, mice were vaccinated in the footpad with 200,000 WT or AIMp1 KO BMDC loaded with B16F10 tumor mRNA and lysate. On day 17, mice were boosted in the footpad with an additional 200,000 BMDC. Tumor sizes were measured by caliper (*n* = 5). Data are displayed as mean ± SEM. **p* < 0.05, ***p* < 0.01, ****p* < 0.001 as determined by paired two-way analysis of variance with Bonferroni *post hoc* test for multiple comparisons.

Figure S2 Characterization of wild-type (WT) and AIMp1KO BMDC populations by flow cytometry indicates no significant differences in subset or lineage marker expression. WT and AIMp1KO BMDCs are differentiated in GM-CSF (20 ng/mL) and IL-4 (10 ng/mL) for 6 days then treated accordingly for 2 days prior to flow cytometry analysis. Cells are gated on the CD11c^+^MHC-II^+^ population and analyzed for CD11b, MHC class II (I-A^b^), CD8α, and CD103 expression.

Figure S3 AIMp1 deficiency does not alter lymphocyte populations in secondary lymphoid organs nor dendritic cells independent T_H_1 differentiation. **(A)** T-cell (CD3^+^NK1.1^−^, CD4^+^CD3^+^NK1.1^−^, and CD8^+^CD3^+^NK1.1^−^), B-cell (CD19^+^), and NK cell (CD3^−^NK1.1^+^) ratios in WT or AIMp1 KO spleens analyzed by flow cytometry (*n* = 3, biological repeats). **(B,C)** WT or AIMp1 KO CD4^+^ T-cells were cultured under T_H_0 or T_H_1 differentiating conditions for 3 days, stimulated overnight with PMA/ionomycin, and treated with Brefeldin A for intracellular staining of IFN-γ (**B**) and RT-PCR of *ifng* transcripts **(C)** (*n* = 3, biological repeats). Data are displayed as mean ± SEM. No significant differences as determined by Student’s two-tailed *t*-test.

Figure S4 Effect of p38 MAPK and PP2A inhibitors on MAPKAPK2 and p38 phosphorylation. **(A–D)** WT or AIMp1KO BMDCs were left untreated (−) or treated (+) with LPS for 30 min in the absence (−) or presence of p38MAPK inhibitor SB202190 or SB203580 with concentrations indicated in the plot. Cells were harvested for western blot analysis of pMAPKAPK2(T334) and β-actin (endogenous control). DMSO was used as vehicle control in groups without inhibitors. **(B,D)** Relative densitometry quantification of pMAPKAPK2 (T334) from **(A,C)**. **(E–H)** WT or AIMp1 KO BMDC were left untreated (−) or treated (+) with LPS for 30 min in the absence (−) or presence of PP2A inhibitors Okadaic acid or Endothall with concentrations indicated in the plot. Cells were harvested for western blot analysis of p-p38 (T180/Y182) and β-actin (endogenous control). DMSO was used as vehicle control in groups without inhibitors. **(F,H)** Relative densitometry quantification of p-p38 (T180/Y182) from **(E,G)**.

Figure S5 Expression of DUSP1/MKP1 and PP2A subunits in wild-type (WT) and AIMp1 KO BMDC following LPS stimulation. **(A)** WT or AIMp1KO BMDCs were treated with LPS for 0, 10, or 30 min. Cells were harvested for western blotting analysis of DUSP1/MKP1 and PP2A subunits (representative of three independent experiments). **(B–D)** Relative densitometry quantification of PP2A subunit proteins from multiple experiments as in **(A)** (*n* = 5, technical repeats, pooled from three independent experiments). Data are displayed as mean ± SEM. No significant differences between specific groups as determined by two-way analysis of variance with Bonferroni *post hoc* test for multiple comparisons.

Figure S6 Kaplan–Meier analyses of AIMp1 and IFN-γ expression in primary melanoma. Kaplan–Meier plots of overall survival probability in TCGA skin cutaneous melanoma dataset (*N* = 431) divided into tertiles based on the expression of AIMP1 and IFNγ, respectively. The numbers of non-surviving and total patients in each group are indicated in the parentheses. The *p*-value was calculated by Cox regression and log rank test.

Figure S7 Full unedited gel image of main figures. **(A)** Figure 2C; **(B)** Figure 3B; **(C)** Figure 3C. All lanes and proteins of interests were highlighted by red arrows and correspond to main figure western blotting images.

Figure S8 Full unedited gel image of supplement figures. **(A)** Figure S4 (top: Figure S4A; bottom: Figure S4B); (**B**) Figure S5. All lanes and proteins of interests were highlighted by red arrows and correspond to main figure western blotting images.
