# Supplementary figures and images for: AIMp1 Potentiates TH1 Polarization and Is Critical for Effective Antitumor and Antiviral Immunity
Source: Front Immunol. 2018 Jan 15;8:1801. doi: 10.3389/fimmu.2017.01801 (PMC5775236; doi:10.3389/fimmu.2017.01801)

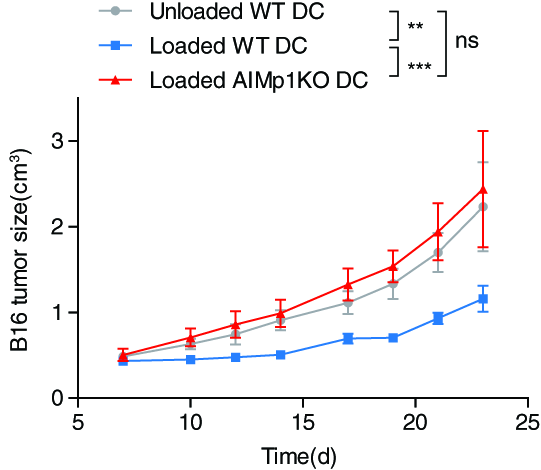

Supplement: Figure S1 — AIMp1 within BMDC promotes control of B16F10 melanoma. Wild-type (WT) C57BL/6:129 F1 animals were challenged with 50,000 B16F10 melanoma tumor cells s.c. on day 0. On day 7, mice were vaccinated in the footpad with 200,000 WT or AIMp1 KO BMDC loaded with B16F10 tumor mRNA and lysate. On day 17, mice were boosted in the footpad with an additional 200,000 BMDC. Tumor sizes were measured by caliper (n = 5). Data are displayed as mean ± SEM. *p < 0.05, **p < 0.01, ***p < 0.001 as determined by paired two-way analysis of variance with Bonferroni post hoc test for multiple comparisons. [file image_1.tif]

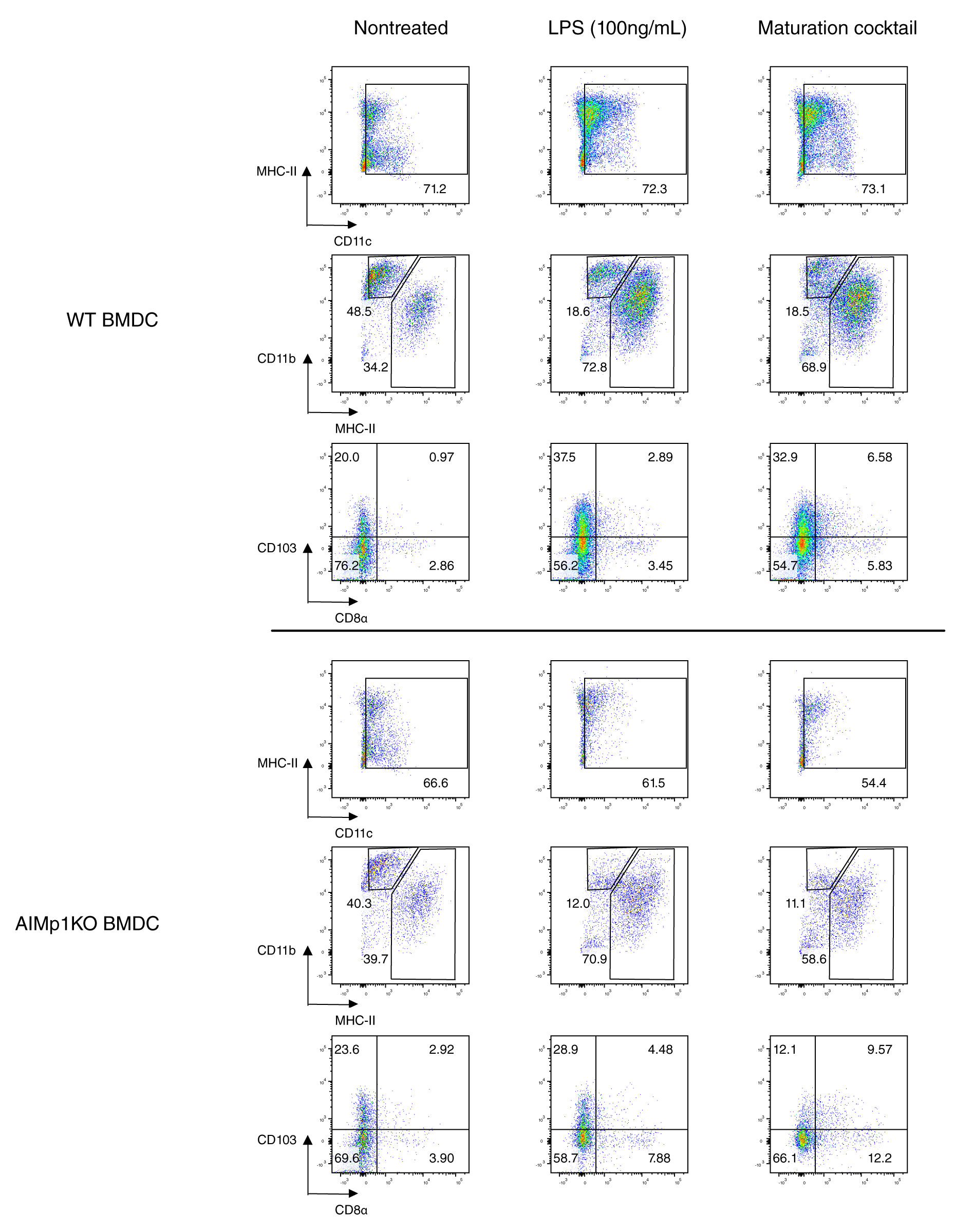

Supplement: Figure S2 — Characterization of wild-type (WT) and AIMp1KO BMDC populations by flow cytometry indicates no significant differences in subset or lineage marker expression. WT and AIMp1KO BMDCs are differentiated in GM-CSF (20 ng/mL) and IL-4 (10 ng/mL) for 6 days then treated accordingly for 2 days prior to flow cytometry analysis. Cells are gated on the CD11c+MHC-II+ population and analyzed for CD11b, MHC class II (I-Ab), CD8α, and CD103 expression. [file image_2.tif]

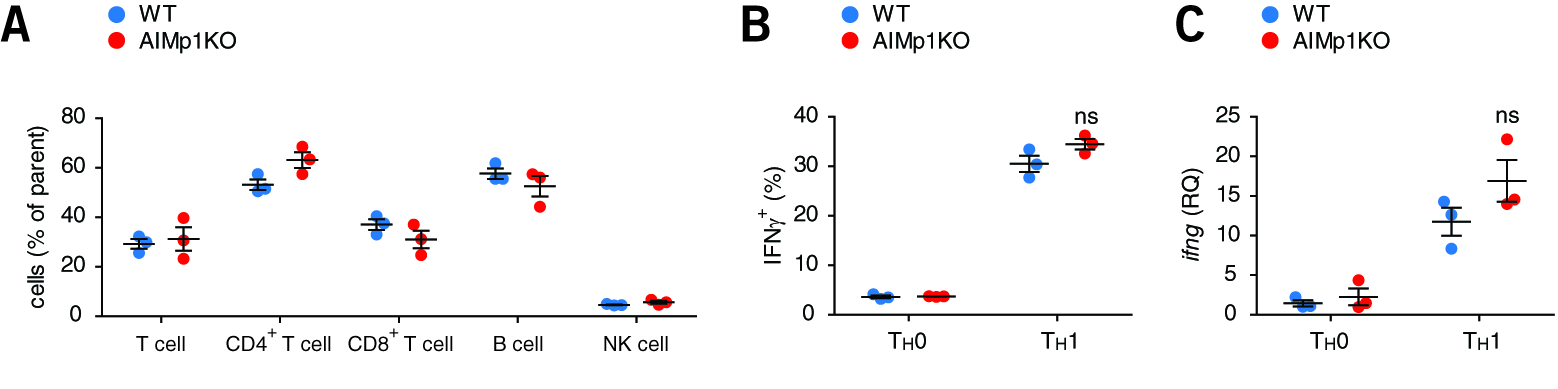

Supplement: Figure S3 — AIMp1 deficiency does not alter lymphocyte populations in secondary lymphoid organs nor dendritic cells independent TH1 differentiation. (A) T-cell (CD3+NK1.1−, CD4+CD3+NK1.1−, and CD8+CD3+NK1.1−), B-cell (CD19+), and NK cell (CD3−NK1.1+) ratios in WT or AIMp1 KO spleens analyzed by flow cytometry (n = 3, biological repeats). (B,C) WT or AIMp1 KO CD4+ T-cells were cultured under TH0 or TH1 differentiating conditions for 3 days, stimulated overnight with PMA/ionomycin, and treated with Brefeldin A for intracellular staining of IFN-γ (B) and RT-PCR of ifng transcripts (C) (n = 3, biological repeats). Data are displayed as mean ± SEM. No significant differences as determined by Student’s two-tailed t-test. [file image_3.tif]

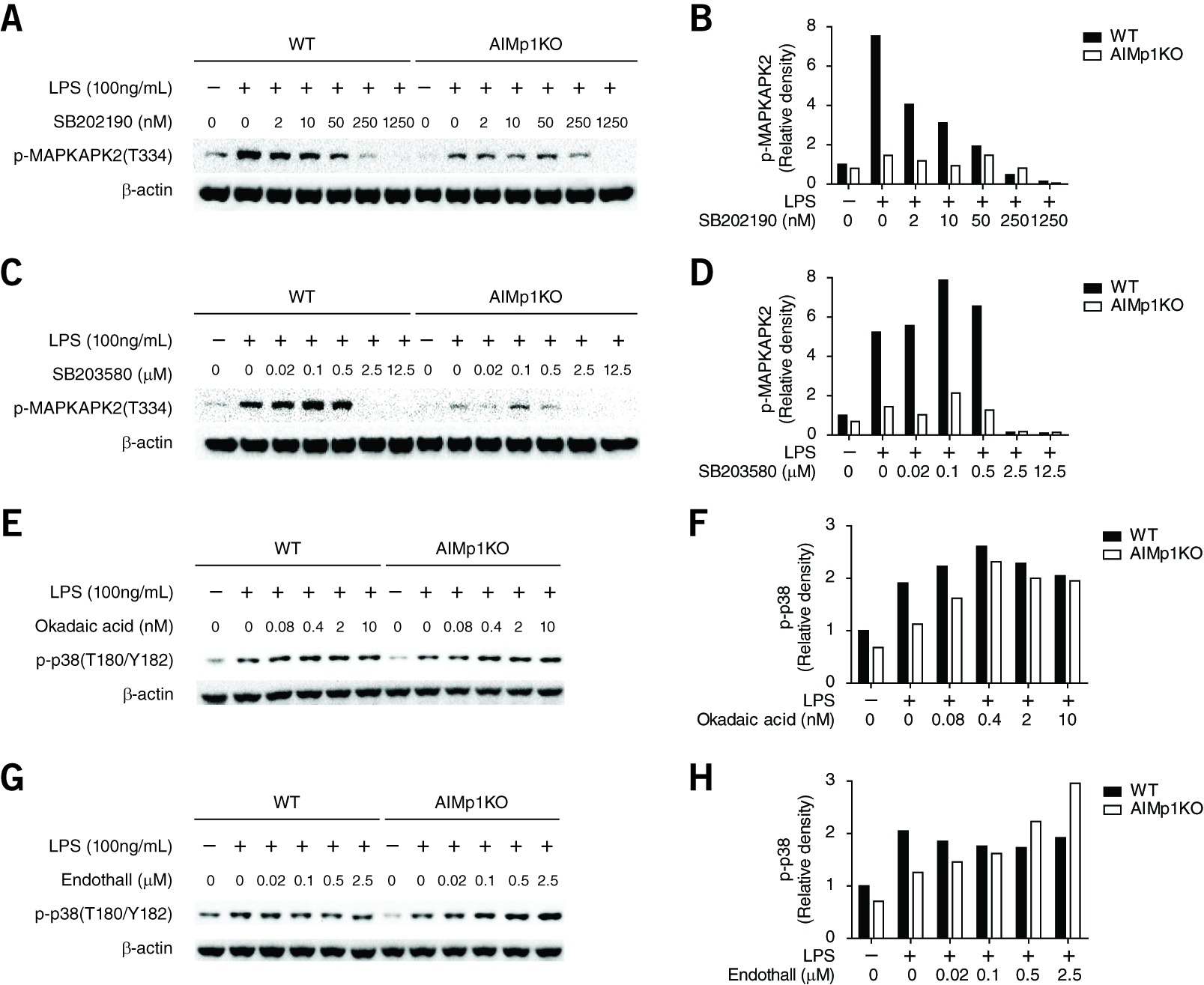

Supplement: Figure S4 — Effect of p38 MAPK and PP2A inhibitors on MAPKAPK2 and p38 phosphorylation. (A–D) WT or AIMp1KO BMDCs were left untreated (−) or treated (+) with LPS for 30 min in the absence (−) or presence of p38MAPK inhibitor SB202190 or SB203580 with concentrations indicated in the plot. Cells were harvested for western blot analysis of pMAPKAPK2(T334) and β-actin (endogenous control). DMSO was used as vehicle control in groups without inhibitors. (B,D) Relative densitometry quantification of pMAPKAPK2 (T334) from (A,C). (E–H) WT or AIMp1 KO BMDC were left untreated (−) or treated (+) with LPS for 30 min in the absence (−) or presence of PP2A inhibitors Okadaic acid or Endothall with concentrations indicated in the plot. Cells were harvested for western blot analysis of p-p38 (T180/Y182) and β-actin (endogenous control). DMSO was used as vehicle control in groups without inhibitors. (F,H) Relative densitometry quantification of p-p38 (T180/Y182) from (E,G). [file image_4.tif]

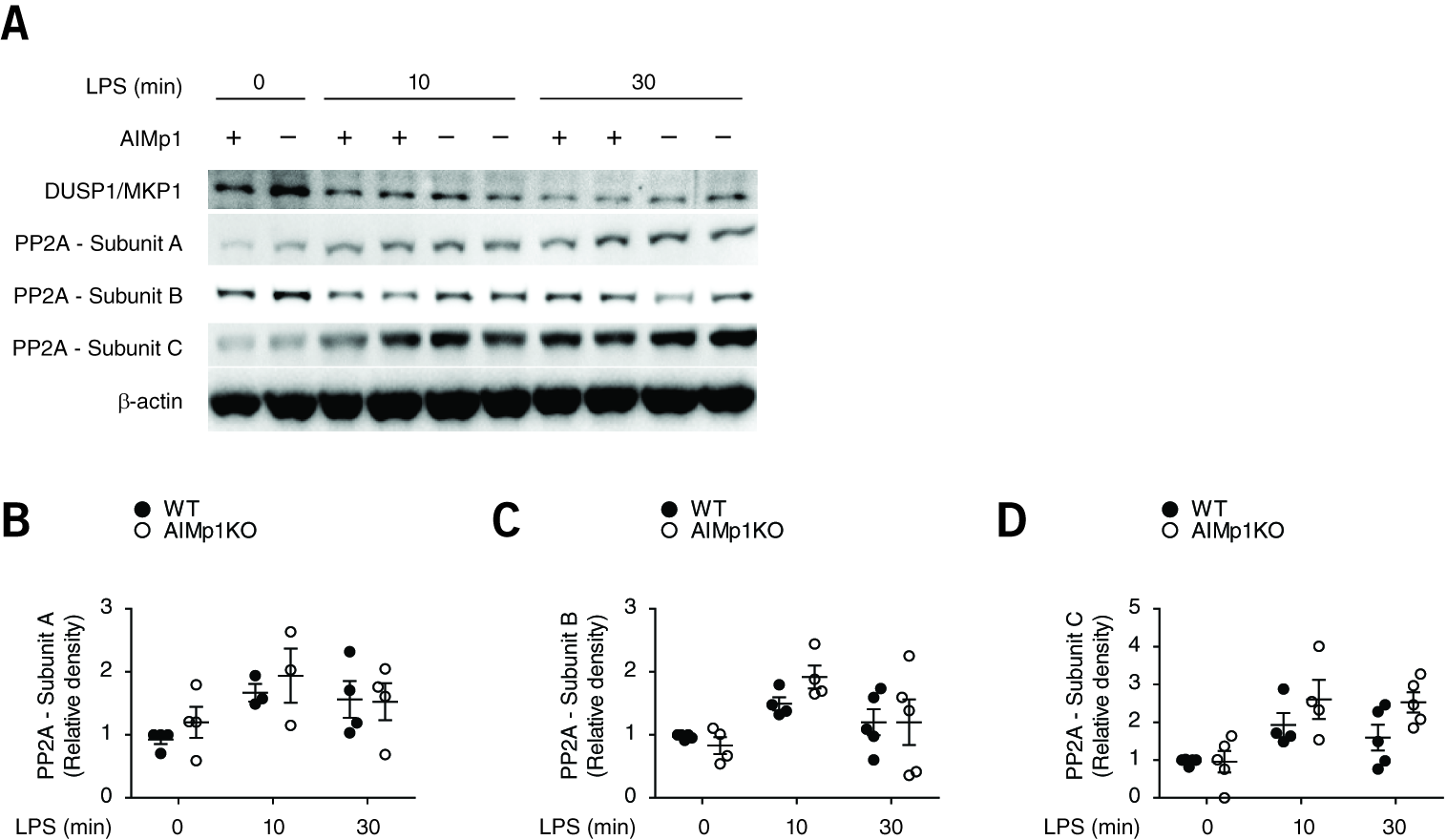

Supplement: Figure S5 — Expression of DUSP1/MKP1 and PP2A subunits in wild-type (WT) and AIMp1 KO BMDC following LPS stimulation. (A) WT or AIMp1KO BMDCs were treated with LPS for 0, 10, or 30 min. Cells were harvested for western blotting analysis of DUSP1/MKP1 and PP2A subunits (representative of three independent experiments). (B–D) Relative densitometry quantification of PP2A subunit proteins from multiple experiments as in (A) (n = 5, technical repeats, pooled from three independent experiments). Data are displayed as mean ± SEM. No significant differences between specific groups as determined by two-way analysis of variance with Bonferroni post hoc test for multiple comparisons. [file image_5.tif]

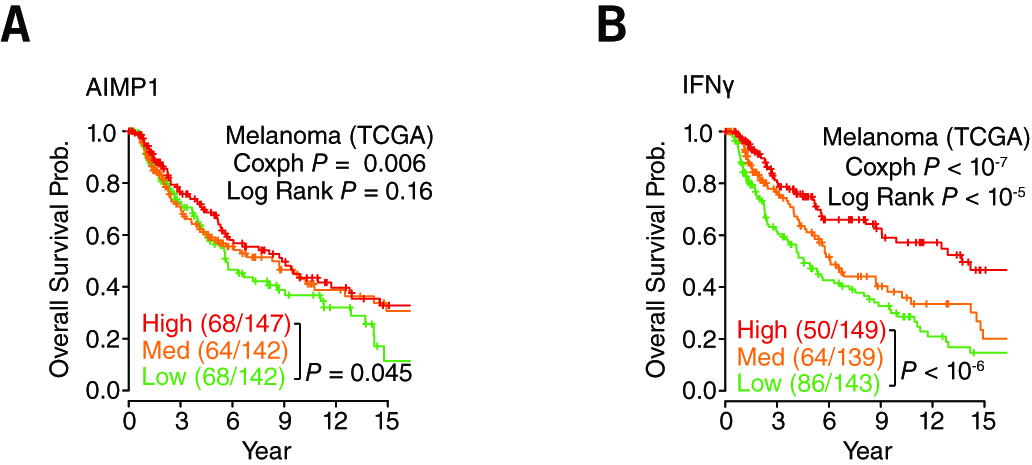

Supplement: Figure S6 — Kaplan–Meier analyses of AIMp1 and IFN-γ expression in primary melanoma. Kaplan–Meier plots of overall survival probability in TCGA skin cutaneous melanoma dataset (N = 431) divided into tertiles based on the expression of AIMP1 and IFNγ, respectively. The numbers of non-surviving and total patients in each group are indicated in the parentheses. The p-value was calculated by Cox regression and log rank test. [file image_6.tif]

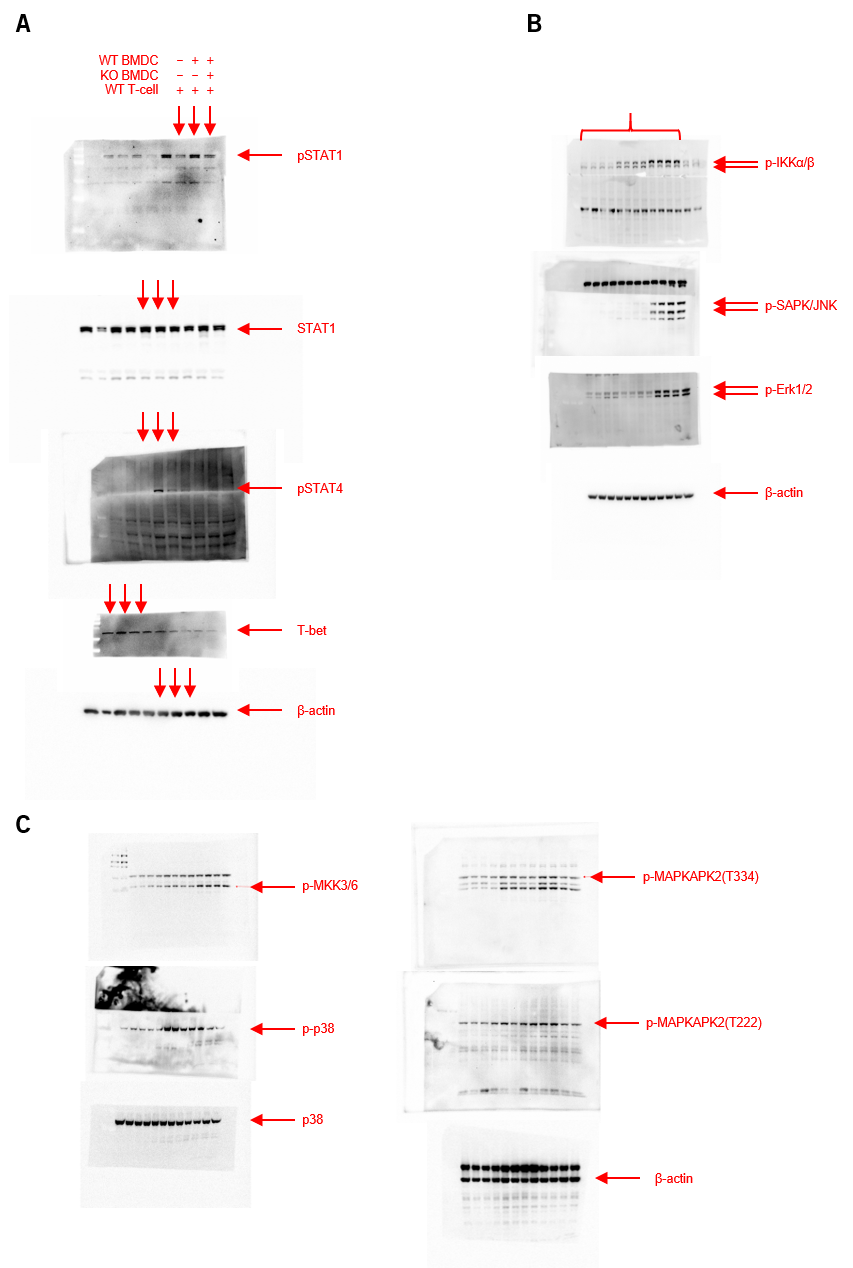

Supplement: Figure S7 — Full unedited gel image of main figures. (A) Figure 2C; (B) Figure 3B; (C) Figure 3C. All lanes and proteins of interests were highlighted by red arrows and correspond to main figure western blotting images. [file image_7.tif]

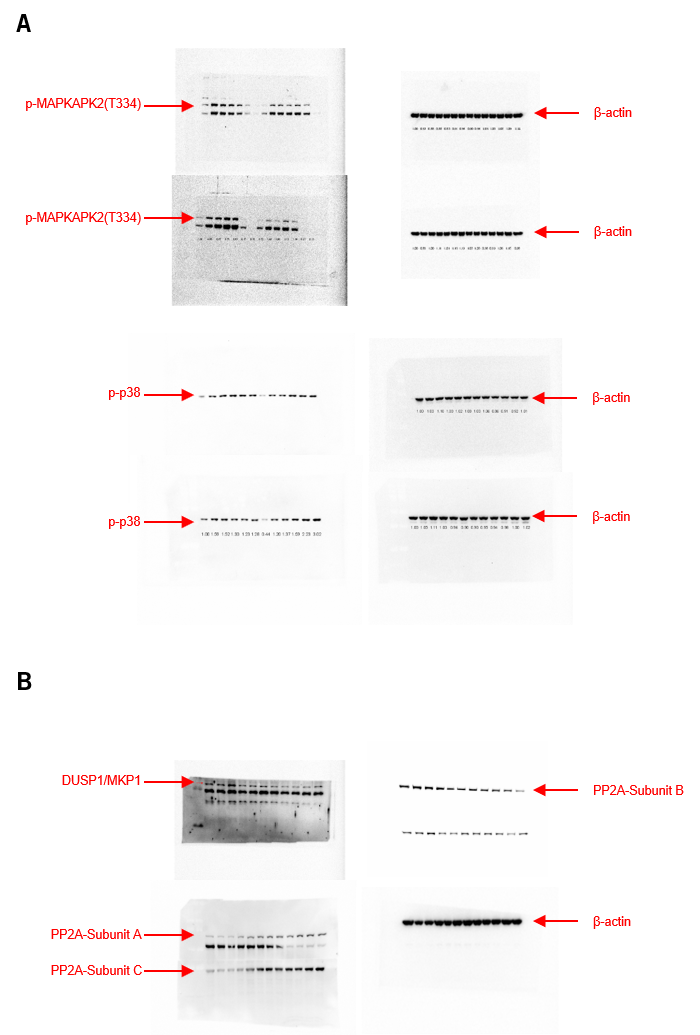

Supplement: Figure S8 — Full unedited gel image of supplement figures. (A) Figure S4 (top: Figure S4A; bottom: Figure S4B); (B) Figure S5. All lanes and proteins of interests were highlighted by red arrows and correspond to main figure western blotting images. [file image_8.tif]
